# Regulated Nuclear Trafficking of rpL10A Mediated by NIK1 Represents a Defense Strategy of Plant Cells against Virus

**DOI:** 10.1371/journal.ppat.1000247

**Published:** 2008-12-26

**Authors:** Claudine M. Carvalho, Anésia A. Santos, Silvana R. Pires, Carolina S. Rocha, Daniela I. Saraiva, João Paulo B. Machado, Eliciane C. Mattos, Luciano G. Fietto, Elizabeth P. B. Fontes

**Affiliations:** Departamento de Bioquímica e Biologia Molecular, BIOAGRO, Universidade Federal de Viçosa, Viçosa, MG, Brazil; University of Basel, Switzerland

## Abstract

The NSP-interacting kinase (NIK) receptor-mediated defense pathway has been identified recently as a virulence target of the geminivirus nuclear shuttle protein (NSP). However, the NIK1–NSP interaction does not fit into the elicitor–receptor model of resistance, and hence the molecular mechanism that links this antiviral response to receptor activation remains obscure. Here, we identified a ribosomal protein, rpL10A, as a specific partner and substrate of NIK1 that functions as an immediate downstream effector of NIK1-mediated response. Phosphorylation of cytosolic rpL10A by NIK1 redirects the protein to the nucleus where it may act to modulate viral infection. While ectopic expression of normal NIK1 or a hyperactive NIK1 mutant promotes the accumulation of phosphorylated rpL10A within the nuclei, an inactive NIK1 mutant fails to redirect the protein to the nuclei of co-transfected cells. Likewise, a mutant rpL10A defective for NIK1 phosphorylation is not redirected to the nucleus. Furthermore, loss of rpL10A function enhances susceptibility to geminivirus infection, resembling the phenotype of *nik1* null alleles. We also provide evidence that geminivirus infection directly interferes with NIK1-mediated nuclear relocalization of rpL10A as a counterdefensive measure. However, the NIK1-mediated defense signaling neither activates RNA silencing nor promotes a hypersensitive response but inhibits plant growth and development. Although the virulence function of the particular geminivirus NSP studied here overcomes this layer of defense in *Arabidopsis*, the NIK1-mediated signaling response may be involved in restricting the host range of other viruses.

## Author Summary

Plants are constantly exposed to microorganisms and, like animals, developed innate immune systems to prevent infections. Although these immune systems protect plants against most potential pathogens, the molecular mechanisms underlying nonhost immunity remain obscure. Here, we describe a novel strategy of plant defenses identified as a target of the geminivirus nuclear shuttle protein (NSP) that suppresses the activity of the transmembrane receptor NIK (NSP-interacting kinase). In addition, we identified a ribosomal protein, rpL10A, as the immediate downstream component of the pathway. Based on our findings, we propose that this pathway is elicited by activation of the receptor NIK1, which results in phosphorylation and translocation of rpL10A to the nucleus. We also provided genetic and biochemical evidence that this regulated trafficking of rpL10A may effectively mount a defense strategy that negatively impacts geminivirus proliferation or movement. Nevertheless, the virulence function of NSP from the bipartite geminivirus CaLCuV (*Cabbage leaf curl virus*) is capable of overcoming the NIK1-mediated defense and thereby enhances the pathogenicity of CaLCuV in *Arabidopsis*. The NIK1-mediated signaling response may be involved in restricting the host range of other viruses.

## Introduction

The obligatory intracellular nature of viruses requires them to make an extensive use of the basic host cellular machinery leading to the development of complex relationships with their biological hosts. Accordingly, research on virus-host interactions has provided considerably insights into basic compatibility functions as well as into the host surveillance mechanisms and the consequent strategies viruses have evolved to overcome the host detection and interdiction systems [1],[2]. In plants, the major described antiviral strategies are the hypersensitive response mediated by resistance genes and, more recently, post-transcriptional gene silencing or RNA interference (RNAi) (for review, see [3],[4]). In the case of geminiviruses, one of the largest and most successful families of plant viruses, the identification of host functions subverted by viral proteins has uncovered novel components from distinct layers of innate host defenses, as adenosine kinase (ADK) and sucrose non-fermenting1 (SNF1) which mediate metabolic defenses possibly linked to gene silencing mechanisms [5]–[7]. Likewise, a novel defense signaling pathway has been identified as virulence target of the bipartite geminivirus nuclear shuttle protein (NSP) [8],[9]. Bipartite geminiviruses (begomoviruses) enhance their pathogenicity by suppressing the kinase activity of the transmembrane receptor-like kinase NIK (NSP-interacting kinase) through NSP-specific binding to the kinase domain.

Geminiviruses constitute a large group of plant viruses with single-stranded DNA genomes that may be organized either in single- or double-component configuration [10]. Typically, both genomic components of bipartite geminiviruses, designated DNA-A and DNA-B, are required for systemic infection. The genes on DNA-A are required for replication, encapsidation and suppression of RNAi defense functions, whereas DNA-B encodes functions required for intra and intercellular movement of viral DNA. DNA-B-encoded NSP facilitates the nucleocytoplasmic traffic of viral DNA and cooperates with the movement protein (MP) to transport the viral DNA to adjacent, uninfected cells [10]–[12]. In addition to interacting with host factors required for basic compatibility functions [13],[14], NSP has also been shown to act as a virulence factor to prevent activation of NIK-mediated response [8].

The NSP partner, transmembrane receptor NIK belongs to the leucine-rich repeats (LRR)-II subfamily of the receptor-like kinase (RLK) family from *Arabidopsis* [15]. Members of this family are organized into a receptor configuration with an N-terminal extracellular domain harboring five LRRs followed by a transmembrane segment and a cytoplasmic C-terminal serine/threonine kinase domain. The members of LRRII-RLKs subfamily has been phylogenetically clustered into three distinct branches of functional relatedness: (i) defense proteins, (ii) developmental proteins, as the somatic embryogenesis receptor kinase (SERK) members and (iii) functionally unassigned proteins [16]. BAK1 (BRI-1 associated kinase), a member of the SERK group II, exhibits independent functions in development and defense as a positive regulator of the plant hormone receptor BRI1 and the resistance gene FLS2 [17],[18],[19],[20]. The NSP-interacting kinase 1, NIK1 (At5g16000), NIK2 (At3g25560), and NIK3 (At1g60800) are inserted into the defense group I of the LRRII-RLK sub-family [16] and they have been initially identified as specific partners of the geminivirus nuclear shuttle protein, NSP [8]. The NSP-NIK interaction is conserved among distinct geminiviral NSPs and NIK homologs from different hosts [8],[9]. Binding of NSP to the NIK activation loop suppresses kinase activity by preventing autophosphorylation of regulatory threonine residues that would otherwise lead to receptor activation [8]. In addition to being inhibited by the viral NSP, loss of NIK function in *Arabidopsis* is linked to an enhanced susceptibility phenotype to infection by a coat protein-less mutant of *Cabbage leaf curl virus* (CaLCuV), suggesting that NIK is involved in antiviral defense responses [8]. In contrast to the resistance function of the related BAK1 gene as a modulator of FGS2 signaling [19],[20], NSP-NIK interaction does not fit into the elicitor-receptor model of resistance [8] and hence the underlying mechanism for a NIK-mediated defense response remains to be deciphered. Here we identified rpL10A (ribosomal protein L10A) as a specific interactor and substrate of the NIK1 receptor. Our study provides both genetic and biochemical evidence that the *rpL10A* gene plays a critical role in a defense strategy as a downstream component of the NIK1-mediated signaling pathway.

## Results

### Identification of Ribosomal Protein as Specific Partners and Substrates for NIKs

To identify potential substrates for NIK1 we performed previously yeast two-hybrid screens with the kinase domain and an *Arabidopsis* cDNA library and we isolated one clone contained a full-length cDNA from the At1g14320 gene which encodes a ribosomal L10 protein (rpL10A) and four clones harboring a full-length *rpL18* cDNA (At2g34480) [21]. In vitro phosphorylation assay with a bacterially expressed GST-fused NIK-kinase domain and GST-fused ribosomal proteins (Figure S1A) demonstrated that NIK1 phosphorylates efficiently rpL10A but not rpL18. The rpL10A protein is a component of the large (60S) ribosomal subunit, which is encoded by a small gene family represented by three copies in the *Arabidopsis* genome (rpL10A, rpL10AB (AT1G26910), and rpL10AC (AT1G66580)) [22]. These rpL10 paralogs share 90–95%, sequence identity among them. They also share significant conservation of primary structure with ribosomal protein L10 from other higher plants, such as rice rpL10 (83% identity; as for NP_001054759), and from other eukaryotic organisms, such as rat rpL10 (67% identity; Q6PDV7, for example), yeast rpL10 (62%; NP_013176) and members of the human QM gene family (69% identity, accession P27635).

We also examined whether rpL10A served as a substrate for other NIK1-related LRRII-RLK proteins (Fontes et al., 2004). NIK2, the NIK1 most related protein, also phosphorylated rpL10A although with less efficiency. In contrast, phosphorylation of rpL10A by NIK3 was barely detectable (data not shown) and the developmental protein SERK1 did not recognize rpL10A or rpL18 as substrates. rpL10A also does not serve as a substrate for either BAK1 or BRI-1 receptors (data not shown). These *in vitro* results implicated rpL10A as a potential, specific substrate for the NIK defense proteins.

### NIK1 Phosphorylates rpL10A and Relocates the Cytosolic Protein to the Nucleus

**Figure 1:**
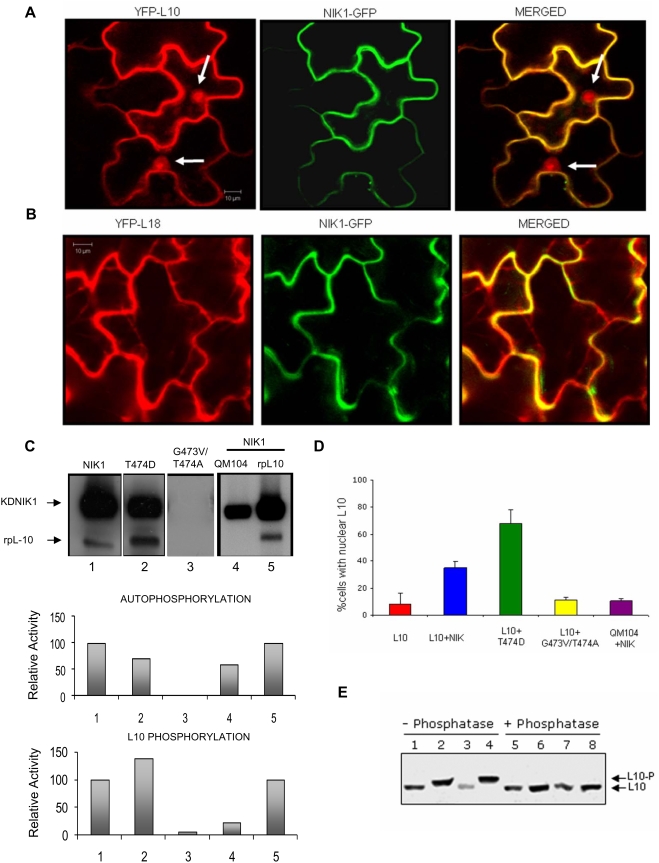
NIK1 phosphorylates rpL10A and relocates the cytosolic protein to the nucleus of transfected cells. (A) Ectopic expression of NIK1 alters the nucleocytoplasmic shutting of rpL10A. YFP-L10 and NIK1-GFP were co-expressed in tobacco leaf epidermal cells, and the subcellular localization of the fluorescent fusion proteins was monitored by confocal microscopy. The frequency of co-transfected cells with rpL10A localized within the nuclei was increased. Full arrows indicate fluorescent nuclei. Scale bars are 10 µm. (B) Confocal microscopy of YFP-L18 and NIK1-GFP co-expressing epidermal cells. YFP-rpL18 and NIK1-GFP were co-expressed in tobacco leaf epidermal cells. Full arrows indicate fluorescent nucleoli. (C) In vitro phosphorylation activity of mutant NIK1s. GST fused to the C-terminal kinase domain (amino acids 298–638) of normal NIK1 (NIK1) or to mutant NIK1s (T474D and G473V/T474A) were produced in *E. coli* and affinity-purified using GST-Sepharose (Figure S2C). Purified GST fusions (as indicated) were incubated with equal amounts of GST-L10 or GST-QM104 in the presence of [γ-^32^P]ATP and separated by SDS-PAGE. The gels were stained with coomassie-blue (not shown) and visualized by autoradiography using a phosphoimager (top panel). The relative activity of autophosphorylation and phosphorylation of the L10 substrate was quantified and expressed as a percentage of the wild type kinase activity. The autophosphorylation activity was expressed as Vunits/µg enzyme/min, and substrate phosphorylation activity as Vunits/µg enzyme/µg substrate/min. (D) The efficiency of rpL10A relocalization to the nuclei correlates with kinase activity of NIK1. Tobacco leaves were co-agroinfiltrated with YFP-L10 and GFP-fusions, as indicated. The percentage of co-transfected cells containing YFP-L10 fluorescence over the nucleoplasm was registered. Values are the mean±SD of three determinations from independent experiments. In each experiment, a total of 100 to 150 cells were observed. (E) Ectopic expression of active NIK1 promotes accumulation of phosphorylated rpL10A in the nuclear extracts of co-transfected epidermal leaf cells. Nuclear extracts were prepared from YFP-rpL10A–transfected leaves (lanes 1 and 5), as well as from leaves co-transfected with YFP-rpL10A and NIK1-GFP (lanes 2 and 6), YFP-rpL10A and G473V/T474A-GFP (lanes 3 and 7), or YFP-rpL10A and T474D-GFP (lanes 4 and 8), separated by SDS-PAGE and immunoblotted using a GFP antibody. In lanes 5, 6, 7, and 8, the nuclear extracts were treated with alkaline phosphatase prior to electrophoresis.

*Arabidopsis* rpL10A is closely related to the human putative tumor suppressor *QM* (69% identity) and to *Jif-1* (Jun interacting factor) that inhibits Jun-Jun dimer formation [23]. These extraribosomal functions of rpL10A are associated with a nuclear localization of the protein. To determine the subcellular localization of the *Arabidopsis* rpL10A homolog, we utilized an *Agrobacterium*-mediated transient expression assay in epidermal cells of tobacco leaves (Figure S1). Confocal microscopy revealed that rpL10A fused to either GFP or YFP localizes predominantly in the cytoplasm but also resides in the nuclei of a small fraction (3%) of transfected leaf cells in which intense rpL10A-GFP or YFP-rpL10A fluorescence was observed over the nucleoplasm (Figure S1C). Immunoblots of nuclear fractions from tobacco leaves expressing rpL10A-GFP further confirmed that rpL10A-GFP also localizes in the nucleus (Figure 1E). This nuclear localization pattern of rpL10A was distinct from the nucleoli-localized fluorescence resultant from expression of rpL18-GFP or YFP-rpL18 fusions (Figure S1D), which served as markers for ribosome assembly that has been shown to occur in the nucleoli [24]. In fact, the nucleolar fluorescence pattern and cytoplasmic localization of GFP-rpL18 and rpL18-YFP control proteins were identical to those observed for the *Arabidopsis* ribosomal proteins rpL23aA and rpL23aB when transiently expressed in tobacco epidermal cells as GFP fusions [25].

Figure S1B also shows the localization of GFP and YFP when expressed alone. As both control proteins are localized in the cytoplasm and also in the nucleus they served as nuclear and cytosolic markers in our assay. Due to the large vacuoles of leaf epidermal cells, the cytoplasm is pushed up against the plasma membrane and fluorescence is restricted to a narrow band (lining the plasma membrane) in medial optical sections in mature leaf cells. This effect is less pronounced with optical slices towards the top of the cells and the cytoplasm is more obvious in tangential optical sections at the outer cell surface [13] (Figure S1B, GFP (top cells); Figure S1C, YFP-L10, top panel).

As a ribosomal protein, the localization of rpL10A over the nucleoplasm may reflect an extraribosomal function of the protein. Accordingly, ectopic expression of NIK1-GFP (Figure 1A) and NIK2-GFP (data not shown) in tobacco leaves altered the nucleocytoplasmic shutting of rpL10A because the cell frequency with nucleus-localized YFP-rpL10A was significantly increased to 38% of co-transfected leaf cells (the yellow pattern in merged image ensured co-expression of the fused proteins in the same cell). The NIK1-mediated nuclear relocalization of ribosomal protein was an rpL10A-specific process as it did not alter the subcellular localization of the ribosomal L18 control protein (Figure 1B). Although both ribosomal proteins were found to associate with the kinase domain of NIK1 in yeast, only rpL10A served as a NIK1 substrate *in vitro*. Likewise, the related SERK1 protein did not recognize rpL10A as substrate (Figure S1A) and did not alter the subcellular localization of the protein (data not shown).

To investigate the significance of rpL10A phosphorylation by NIK1 *in vivo* and its possible link to the NIK1-mediated nuclear relocalization of the ribosomal protein, we co-expressed an YFP-rpLl10 fusion with either a constitutively hyperactive NIK1 mutant, T474D, in which the regulatory phosphorylation site Thr-474 was mutated to an aspartate (Figure 1C, lane 2; Figures S2A and S2C) or an inactive form of NIK1, G473V/T474A mutant (Figure 1C, lane 3). The subcellular localization of rpL10A in co-transfected leaf cells was assayed by confocal microscopy and biochemical fractionation. Co-expression of YFP-rpL10A and NIK1-GFP redirected rpL10A from the cytoplasm to the nucleus in 38% of co-transfected *N. tabacum* leaf cells, confirming the previous results (Figure 1D). However, the efficiency of NIK1-mediated rpL10A nuclear localization was increased to 68% of transfected cells when YFP-rpL10A was co-expressed with the hyperactive T474A mutant. This *in vivo* result correlated well with the *in vitro* rpL10A phosphorylation activity of T474D mutant which was 1.5 fold higher than that of NIK1 (Figure 1C, lane 2). In contrast, the inactive G473V/T474A mutant failed to redirect rpL10A to the nuclei of co-transfected cells (Figure 1D). These results indicate that NIK1-mediated nuclear relocation of rpL10A is dependent on its kinase activity. We also performed the reverse experiment with a mutant rpL10A (QM104, Figures S2B and S2C) in which the replacement of a serine residue, position 104, to alanine blocked NIK1 phosphorylation (Figure 1C, lane 4) and hence impaired NIK1-mediated nuclear redirection of the mutant rpL10A protein (Figure 1D, QM104).

To further confirm that the translocation of rpL10A to the nuclei was driven by phosphorylation, we directly monitored the presence of rpL10A phosphoproteins in the nuclei of NIK1, T474D or G473V/T474A co-transfected tobacco leaf cells by immunoblotting of nuclear extracts (Figure 1E). In YFP-rpL10A-expressing tobacco leaf cells, a small fraction of hypophosphorylated rpL10A accumulated in the nuclei of transfected cells (lane 1). However, ectopic expression of *NIK1* increased the nuclear concentration of YFP-rpL10A proteins as a slower migrating electrophoretic band (lane 2), which was reversed to the normal lower YFP-rpL10A band by alkaline phosphatase treatment of nuclear extracts (compare lanes 2 and 6). This result indicates that the observed change in electrophoretic migration of the YFP-fusion was due to phosphorylation. In support of this, co-expression of rpL10A with an inactive NIK1 (G473V/T474A) did not change the normal electrophoretic pattern of the nuclear localized YFP-rpL10A (lane 3) and hyperactive NIK1 (T474D) intensified the accumulation of nuclear phosphorylated rpL10A (lane 4). The increased accumulation of phosphorylated rpL10A-YFP and GFP-rpL10A in the nucleus mediated by NIK1 expression was not an apparent effect on protein stability due to phosphorylation because rpL10A when expressed alone or with NIK, as well as the mutant QM104, which is not phosphorylated by NIK1, accumulated to similar extent in agroinoculated tobacco leaves (data not shown and Figure S3). Collectively, these results indicate that phosphorylation of rpL10A by NIK1 relocates the protein from the cytoplasm to the nucleus.

**Figure 2:**
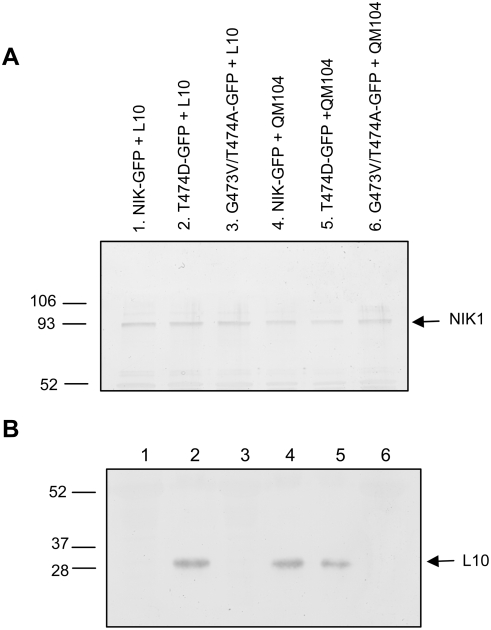
NIK1 associates detectably with rpL10A *in vivo*. Tobacco leaves were agroinoculated carrying the DNA constructs as indicated on the top of the lanes. About 72 hours post-transfection, protein extracts were prepared from protoplasts of agroinoculated leaves and used for isolation of protein complexes with anti-GFP serum and protein A-Sepharose. Immunocomplexes were separated by SDS-PAGE and probed with either GFP antibody (A) or an anti-rpL10A serum (B).

Because interaction of NIK1 with its substrate rpL10A is difficult to detect *in vivo* by co-immunoprecipitation, probably due to its transient nature along with the pre-requisite of receptor activation, we anticipated that overexpression of the constitutively active T474D mutant receptor and mutant rpL10A (QM104) in our transient co-expression assays would facilitate *in vivo* detection of NIK1-rpL10A complex (Figure 2B). Accordingly, while rpL10A could be only detected in complexes formed with T474D (lane 2), the rpL10A mutant associated detectably *in vivo* with both NIK1 (lane 4) and T474D (lane 5). In contrast, neither intact rpL10A nor mutant rpL10A (QM104) interacted with the inactive G473V/T474A mutant receptor (lanes 3 and 6), which in fact was not expected to assume a proper conformation for substrate binding due to the lack of autophosphorylation activity (Figure 1D). This was not the case for NIK1 because we could not detect NIK1-rpL10A complex formation under the conditions of our experiment but we did detect binding between NIK1 and QM104. The complex formed between NIK1 and QM104 is expected to be sufficiently stable to be detected in our assay because QM104 cannot be phosphorylated by NIK1 (Figure 1D). In addition to demonstrating *in vivo* interaction between rpL10A and NIK1, these results also confirmed that intact and mutant NIK1s accumulated at similar levels in our transient expression assay (Figure 2A), further substantiating the argument that relocation of rpL10A to the nucleus is dependent on NIK1 kinase activity.

### Loss of *rpL10A* Function Enhances Susceptibility to Geminivirus Infection Resembling the *nik1* Phenotype

**Figure 3:**
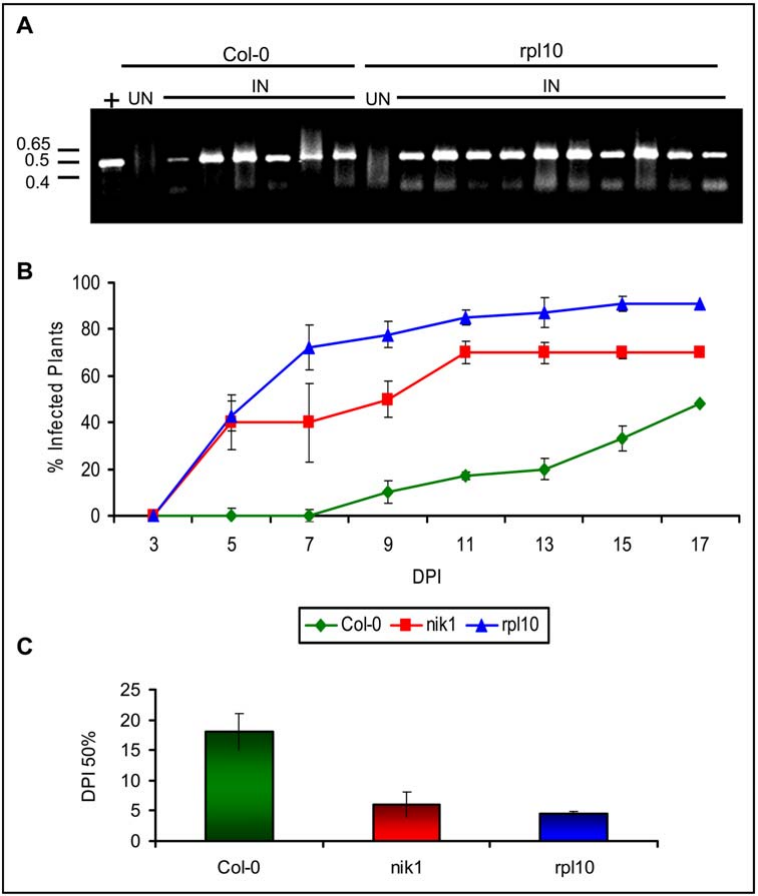
*rpL10A* knockout lines display enhanced susceptibility phenotype to geminivirus infection as *nik1* null alleles. (A) Detection of viral DNA in infected lines. Ecotype Col-0, *nik1*, and *rpl10a* lines at the seven-leaf stage were infected with an attenuated form of CaLCuV by biolistic delivery of tandemly repeated viral DNA-A and DNA-B. Total DNA was isolated from infected plants at 7 DPI, and viral DNA was detected with DNA-B-specific primers. IN refers to CaLCuV-inoculated plants and UN to mock-inoculated plants. + indicates control plasmid DNA as template. The positions of DNA standard markers are shown on the left in kbp. The gel shows a representative sampling of Col-0 and *rpL10A* infected plants. (B) Course of infection in *rpl10a*, *nik1*, and Col-O lines. Values represent the percentages of systemically infected plants at different days postinoculation (DPI). The data are the means of three independent experiments. In each experiment, 20 plants of each line were inoculated with 2 µg of tandemly repeated DNA-A plus DNA-B per plant. (C) Infection rates in *rpl10a* and in *nik1* KO lines. The infection rate is expressed as number of DPI required to get 50% infected plants (DPI^50%^). Values for DPI^50%^ are the mean±standard deviation from five replicas.

To investigate the possibility that the nuclear hyperphosphorylated rpL10A might play an extraribosomal role in NIK1-mediated signaling response, we compared the susceptibility of *nik1* [8] and *rpl10a* null alleles (Figure S4) [21] to geminivirus infection (Figure 3). We have demonstrated previously that removal of coat protein (CP) sequences from CaLCuV DNA-A attenuated the virus in wild type Col-0 plants but not in the *nik1* null background [8]. Likewise, a CP null mutant, which was generated by introducing a premature stop codon in CP sequences, attenuated the virus in wild type and *rpl18* knockout lines, but not in *rpl10a* mutant lines [21]. Here, we directly compared the susceptibility phenotype to geminivirus infection of *rpl10a* (Figure S4C) with that of *nik1* lines by inoculating Col-0 plants as well as *nik1* and *rpl10a* mutant lines with an attenuated *CP* null mutant of CaLCuV [21]. The accumulation of viral DNA was detected in all symptomatic plants by PCR with viral DNA-specific primers (Figure 3A). Loss of *rpL10A* function recapitulated the *nik1* enhanced susceptibility phenotype to geminivirus infection, as the *rpl10a* knockout lines developed similar severe symptoms and displayed similar infection rate as *nik1* (Figure 3B). In other independent experiments, the infectivity data, expressed as DPI^50%^ (days postinoculation to reach 50% of infected plants), further confirmed that *rpl10a* and *nik1* displayed the similar enhanced efficiency of virus infection as compared to Col-0 (Figure 3C). These results together with our biochemical data genetically linked the *rpL10A* gene to NIK1-mediated signaling pathway.

### Geminivirus Infection Interferes with the NIK1-Mediated Nuclear Relocalization of rpL10A

**Figure 4:**
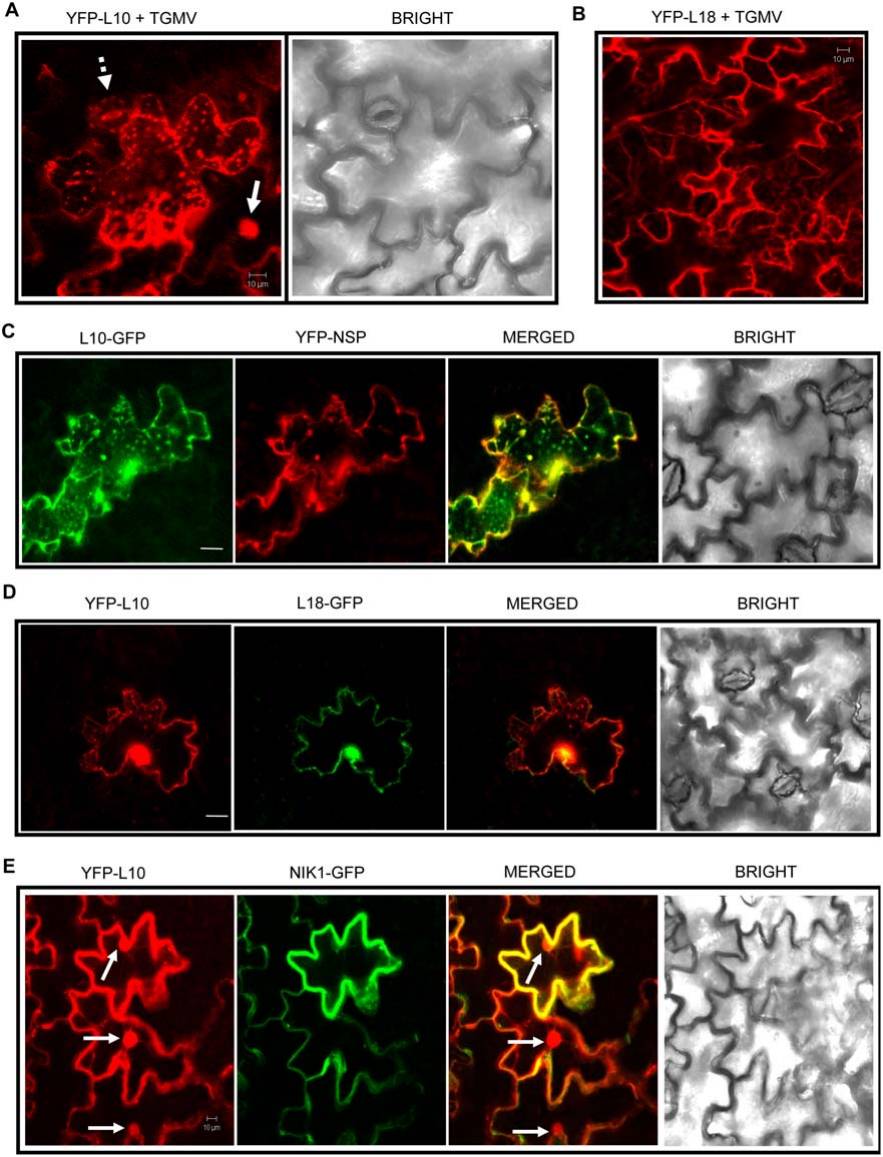
Geminivirus infection interferes with the NIK1-mediated nuclear localization of rpL10A. Tandemly repeated TGMV DNA-A and DNA-B were introduced into tobacco leaves by biolistic inoculation. Five days postinoculation, the infected leaves were co-agroinfiltrated with the combinations: YFP-L10 (A), YFP-L18 (B), L10-GFP and YFP-NSP (C), YFP-L10 and L18-GFP (D), YFP-L10 and NIK1-GFP (E). Full arrows indicate fluorescent nuclei, and traced arrows stomata. Scale bars are 10 µm. Bright is the corresponding transmitted light image of tobacco leaf epidermal cells.

The enhanced susceptibility phenotype to geminivirus infection of *rpl10a* lines may result from pleiotropic effects caused by inactivation of a general translation-controlling ribosomal gene rather than from inactivation of a specific antiviral signaling pathway. To distinguish between these possibilities we attempted to develop a transient infection assay to determine whether geminivirus would interfere with the NIK1-driven nuclear relocalization of rpL10A as a counterdefensive measure. Tobacco leaves were biolistically inoculated with TGMV (Tomato golden mosaic virus) prior to agroinoculation with YFP-rpL10A fusion and the localization of the ribosomal protein was monitored by confocal microscopy of epidermal cells. We chose TGMV because it efficiently infects tobacco (Figures S5A and S5B) and interaction of TGMV NSP with NIKs is not host-specific and hence it is expected to suppress the kinase activity of both endogenous tobacco NIK and ectopically expressing *Arabidopsis* NIK1 [8],[9],[26]. We showed here that geminivirus infection promoted mislocalization of YFP-rpL10A causing it to accumulate as dispersed punctate bodies in the cytoplasm of leaf cells (Figure 4A). In contrast, the intracellular localization of the ribosomal L18 control protein was not altered in epidermal cells of TGMV-infected leaves (Figure 4B).

The low geminivirus infection efficiency in epidermal cells requires the infected cells to be individually identified in order to precisely associate an induced subcellular change with geminivirus infection. We took advantage of the nucleocytoplasmic shuttling properties of the geminivirus NSP to develop a molecular marker for infected cells [13],[27]. Like other geminivirus nuclear shuttle proteins, NSP accumulates within the nuclei of NSP-expressing tobacco leaf cells (Figure S5C). However, in infected cells the presence of geminivirus MP relocates YFP-NSP to the cell periphery. Thus, in our transient infection/expression assay, the cytosolic localization of NSP was an efficient indicator of TGMV-infected cells (Figure S5C). Using NSP as marker, we confirmed that the formation of punctuate YFP-rpL10A bodies was induced by geminivirus infection. In fact, the appearance of this abnormally rpL10A aggregates was restricted to the cytoplasm of TGMV-infected cells, as judged by co-localization with a host protein cytosolic marker (data not shown) and the viral NSP (Figure 4C; compare yellow and green patterns in merged image). The TGMV-induced rpL10A punctate corpuscles neither represent aberrant ribosomes because a ribosomal protein L18 marker did not co-localize with them (Figure 4D) nor are a result of cell death or lysis (Bright field). The inclusion of YFP-L18 (Figure 4B) and L18-GFP (Figure 4D) fusions in the assay also served as negative controls, confirming that TGMV-induced aggregates were specific for either YFP-rpL10A or rpL10A-GFP. More likely, the abnormally dispersed cytosolic rpL10A bodies are formed as a result of suppression of NIK1 function by geminivirus NSP binding that prevents rpL10A phosphorylation. In support of this, overexpression of *Arabidopsis* NIK1 restored the wild type localization of rpL10A as expected from titration of the virally produced NSP inhibitor by a molar excess of ectopically expressed NIK1 (Figure 4E, compare yellow and red patterns in merged image). These results are consistent with the *in vitro* stoichiometry of NIK1 inhibition by NSP (Figure S6) [8]. To test further this hypothesis NIK1 was overexpressed under the constitutive CaMV 35S promoter in tomato plants for gain-of-function analysis (see below).

### Ectopic Expression of NIK1 in Tomato Attenuates Symptom and Delays Begomovirus Infection

**Figure 5:**
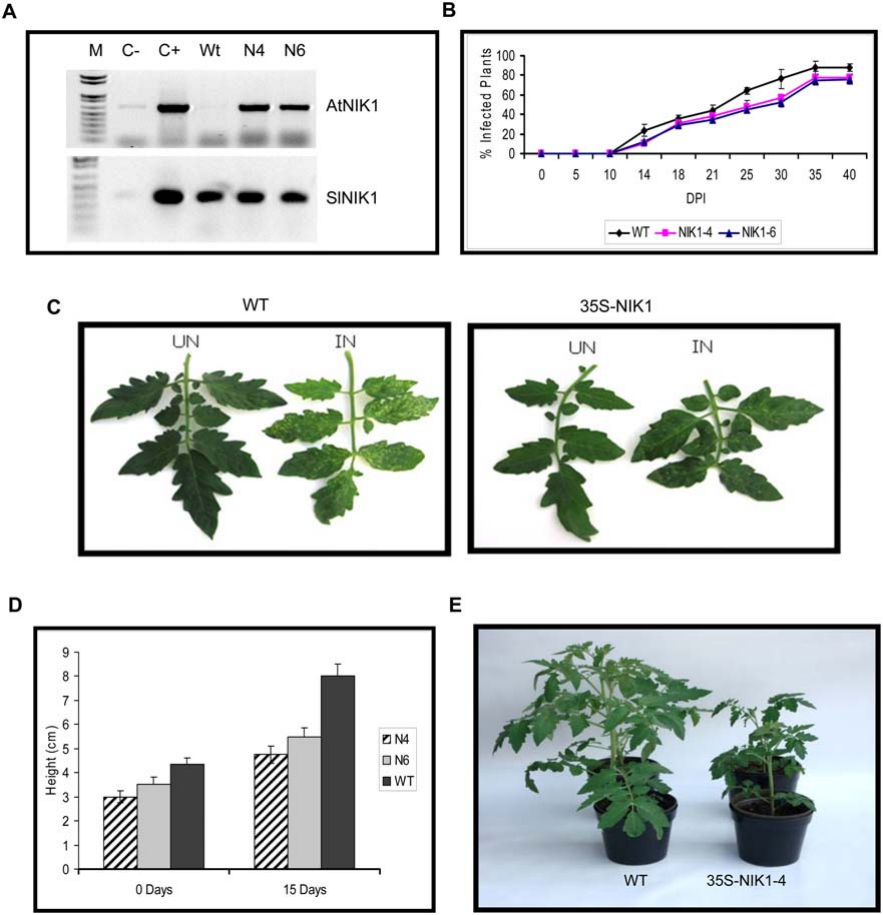
Overexpression of NIK1 in tomato delays virus infection and impacts development. (A) NIK transcript accumulation in transgenic lines. Semi-quantitative RT–PCR was performed with cDNA prepared from WT seedlings, 35S:NIK1-4 (N4) or 35S:NIK1-6 (N6) transgenic lines with gene-specific primers, as indicated. Control reactions were conducted with polyA+ RNA from WT and transgenic cell lines without reverse transcriptase (C−) and with plasmid DNA (C+). (B) Symptoms associated with ToYSV infection in WT and 35S:NIK1-4 (35S-AtNIK1) T1 transgenic lines. WT plants and 35S:NIK1-4 (N4) T1 lines at the six-leaf stage were infected with ToYSV by biolistic delivery of tandemly repeated viral DNA-A and DNA-B. UN indicated plants that were bombarded with tungsten particle without viral DNA and IN shows infected plants at 28 days postinoculation. (C) Course of infection in WT and 35S-NIK1-4 lines. WT plants, T1 35S:NIK1-4 (N4), and T1 35S-NIK1-6 (N6) transgenic lines at the six-leaf stage were infected with ToYSV by biolistic delivery of tandemly repeated viral DNA-A and DNA-B. Values represent the percentages of systemically infected plants at different days postinoculation (DPI). The data are the means of three independent experiments. In each experiment, 20 plants of each line were inoculated with 2 µg of tandemly repeated DNA-A plus DNA-B per plant. (D) Growth rate of T0 transgenic (35S-AtNIK1) plants in comparison with untransformed, wild type plants. The growth rate of *in-vitro*-grown seedlings was measured as a function of the height of the plant. To evaluate growth rate, T0 primary transformants 35S-NIK1-4 (N4) and 35S-NIK1-6 (N6) as well as in-vitro–grown wild type (WT) lines were each replicated into ten uniformly sized clones that were allowed to regenerate for one week when height measurements were initiated (Day 0). The height was recorded again after 15 days of growth. (E) Developmental performance of T0 35S-AtNIK1 uninfected transgenic lines and WT plants (WT), 30 days after their transfer to the greenhouse.

NIK was identified initially in tomato (SlNIK) and soybean (GmNIK) by its capacity to interact with NSP from tomato-infecting begomoviruses, demonstrating that interaction with NIK is conserved among begomovirus NSPs [9],[28]. This prompted us to examine whether molar excess of NIK could overcome NSP inhibition by overexpressing NIK1 under the constitutive CaMV 35S promoter in tomato plants (Figure 5A). Typical phenotypes associated with growth inhibition were observed in the NIK1-overexpressing tomato lines. In the first 4 weeks of the plant regeneration process, leaf expansion and regeneration efficiency in the NIK1-overexpressing regenerants were lower than in control, pCAMBIA-transformed regenerants and in wild type. In general, the growth rate of *in-vitro*-regenerated tomato transgenic lines (35S-NIK1-4; N4 and 35S-NIK1-6; N6) was retarded when compared with *in-vitro*-grown wild type plants (Figure 5D). The developmental phenotype of T0 transgenic lines persisted after transferring them to greenhouse and the growth of vegetative parts of tomato transgenic lines was retarded when compared with type plants (Figure 5E). However, stunted growth was less accentuated in greenhouse-grown T0 progenies (T1 plants) and their developmental performance was almost similar to wild type lines (data not shown). Therefore, for infectivity assays, we used kanamycin-resistant NIK1-overexpressing plants from the T1 generation.

Wild type (WT) and NIK1-overexpressing tomato lines in the T1 generation (35S-NIK1-4, N4 and 35S-NIK1-6, N6) were inoculated with the highly pathogenic tomato-infecting begomovirus ToYSV (Tomato yellow spot virus) [29]. The accumulation of viral DNA was detected by PCR and the severity of symptoms was quantified by the intensity of yellow spots per area of infected leaves (Figure 5C). While the wild type plants developed severe symptoms of ToYSV displaying yellow spots all over the leaves (>10 spots/cm^2^), the symptoms in overexpressing lines were attenuated with few yellow spots per leaf (≤2 spots/cm^2^). In addition, overexpression of NIK1 delayed the course of infection and decreased the infection efficiency (Figure 5B). By 30 dpi, 56% (±3.02) of 35S-NIK1-4, 52% (±4.1) of 35S-NIK1-6 lines and 76% (±7.06) of untransformed plants were infected. These results indicate that overexpression of AtNIK1 in tomato attenuates symptom and delays begomovirus infection.

### The NIK1-Mediated Signaling Pathway May Represent a Novel Strategy of Plant Defenses

In plants, the major defense responses against virus consist of RNA silencing and gene-for-gene resistance [30]. In the case of geminiviruses, the viral transcriptional activator protein (TRaP) and AC4/C4 protein have been shown to suppress RNA silencing [31]. Our results indicate that rpL10A plays an important role in plant defense response as a downstream effector of NIK1 signaling. To examine whether L10 would function as a component of the silencing machinery we used a RNA silencing-inducing approach by co-expressing GFP, an inverted repeat GFP RNA as a strong silencing inducer (dsGFP) and a test or control construct in leaf epidermal cells upon agroinfiltration. Expression of GFP was visualized under UV light (Figure S7A) and transcript accumulation was detected by RT-PCR (Figure S7B). As expected, expression of the viral HC-Pro control protein suppressed GFP-directed silencing in the transient system by supporting GFP transcript and protein accumulation in the presence of dsGFP expression. Expression of rpL10A either alone or with NIK1 did not suppress dsGFP-mediated GFP silencing. More importantly, inactivation of the endogenous rpL10A homolog by expression of dsrpL10A (Figure S7B, NbL10 lanes 9 and 10) did not prevent GFP-directed silencing (Figure S7A, panel 10). These results indicate that rpL10A is not likely to function in RNA silencing. We also investigated whether NIK1-mediated nuclear relocalization of rpL10A would trigger a hypersensitive response as expected for a gene-for-gene resistance mechanism [4]. Co-expression of rpL10A and NIK1 or constitutively hyperactive NIK1 in tobacco infiltrated leaves neither caused necrotic lesions nor induced PR genes expression (Figures S8A and S8B), disfavoring an effector-triggered immune response as the basis for NIK1-mediated defense. More likely the NIK1-mediated response represents a novel layer of the innate host defenses that may act on basic compatibility processes to negatively affect virus infection at the expense of normal growth. Accordingly we found that overexpression of *NIK1* in tomato inhibited plant growth and development, a scenario that is likely to impose constraints to virus proliferation and/or spread (Figures 5D and 5E).

## Discussion

Despite extensive studies investigating how viruses influence their hosts, our knowledge about host factors controlling viral disease is still incipient. Here we describe a novel strategy of plant defense response against virus. We propose that this pathway is elicited by activation of the transmembrane receptor NIK1 which results in phosphorylation and translocation of rpL10A to the nucleus. We also provided genetic and biochemical evidence that shuttling of rpL10A may effectively mount a defense strategy that negatively impacts geminivirus proliferation or movement. Firstly, inactivation of *rpL10A* gene enhances geminivirus susceptibility, a phenotype resembling that of *nik1* knockout lines. Furthermore, geminivirus infection interferes with the nucleocytoplasmic shutting of rpL10A and the ribosomal protein forms dispersed punctate corpuscles over the cytoplasm of infected cells. This induced-change in rpL10A subcellular localization may result from suppressing NIK1 kinase activity by the viral NSP protein. NSP inhibits kinase activity of NIK1 by binding to the activation-loop and preventing autophosphorylation of regulatory threonine residue (data not shown). In our infection/expression transient assay in TGMV-infected tobacco leaves, concomitant overexpression of NIK1 restores the normal subcellular localization of YFP-rpL10A, which favors the argument that the produced TGMV NSP inhibitor is titrated out by a molar excess of exogenously introduced NIK1. These results may provide a direct *in vivo* link between NSP virulence and NIK1 defense functions, strongly suggesting that NSP physically associates with and inhibits NIK1 *in vivo* as well. Consistent with this hypothesis overexpression of NIK1 in tomato attenuates symptoms and delays the onset of ToYSV infection. Nevertheless, constitutive expression of *AtNIK1* under the control of 35S promoter in tomato plants also led to growth inhibition under optimal growth conditions.

The virulence function of NSP by targeting and inhibiting the NIK1 kinase activity is likely to enhance the pathogenicity of CaLCuV in *Arabidopsis*. Thus, it is not surprising that the enhanced susceptibility phenotype displayed by single mutants of *rpL10A* or *NIK* alleles can only be monitored by challenging *Arabidposis* with an attenuated form of the virus. In fact, *CP* null mutants of CaLCuV DNA-A delays infection and attenuates symptoms in wild type lines but not in *nik1* or *rpl10a* knockouts in which the coat protein-less mutant promotes wild type-like infection. In bipartite geminiviruses, the coat protein has been demonstrated to act as an auxiliary protein for NSP function by increasing the accumulation of viral ssDNA, the substrate for NSP [32]. Therefore, the coat protein may indirectly stimulate the NSP shuttling function and thereby may increase the efficiency of intracellular viral DNA trafficking, potentiating viral infection. As an inhibitor of NIK kinase activity, the level of NSP inhibition will depend on the ratio of NSP and NIK concentration (Figure S6). Because concentration of viral produced NSP in infected cells will depend on the dosage of accumulated viral DNA, the variation on infection efficiency promoted by wild type or CaLCuV mutants will affect the extent to which NIK-mediated pathway is inhibited. Furthermore, both NIK and rpL10 are encoded by small gene families (three gene copies) whose members may functionally substitute for one another. Functional redundancy of rpL10 family may explain why we did not observe any phenotypic variation between *rpl10a* knockout and wild type lines grown under normal conditions. These observations may also implicate a gene dosage-like effect on NIK-NSP interaction, which may explain at least in part a wild type-like infection promoted by *CP* null mutants in *nik1* and *rpl10a* genetic background. If this is the case, in a triple *rpL10A* mutant, the infection rate by CaLCuV would be enhanced to such extent that would be possible to distinguish it from a relatively lower rate of infection displayed by wild type lines infected by wild type CaLCuV. Nevertheless, the essential character of ribosomal genes [22] is likely to complicate attempts at further pursuing these experiments.

**Figure 6:**
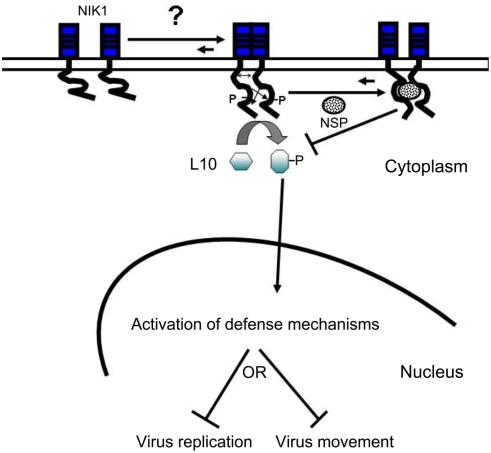
Model for NIK1-mediated signaling pathway. Stress-induced oligomerization of the extracellular domain of NIK1 brings the intracellular kinase domains into proximity and allows them to transphosphorylate and activate one another. Upon activation, NIK1 phosphorylates rpL10A, promoting its translocation to the nucleus where it may mount a defense strategy that prevents virus proliferation of spread. Conversely, binding of NSP to the NIK1 kinase domain (A-Loop) inhibits autophosphorylation of NIK1 and, thus, prevents receptor kinase activation and signaling.

Our current findings together with previous results [8] support a proposed mechanistic model for NIK1-mediated signaling pathway and its interaction with geminiviral protein (Figure 6). In response to unidentified stimuli, the LRR extracellular domain undergoes oligomerization bringing the intracellular kinase domains into close proximity to transphosphorylate and to activate one another. *In vitro* kinetic studies on NIK1 kinase activity and activation provided evidence that oligomerization of NIK1 precedes transphosphorylation and activation of the kinase [8]. The active kinase recruits and phosphorylates the downstream component rpL10A, which results in translocation of the ribosomal protein to the nucleus where it may function to directly mount a defense response that negatively impacts virus replication and/or movement. Alternatively or additionally, as a putative translational control factor, redirecting rpL10A to the nucleus may shut down protein synthesis, thereby impairing virus infection. Counteracting the pathway activation mechanism, binding of NSP to the kinase domain impairs autophosphorylation of regulatory threonine residues within the A-loop and hence prevents activation of NIK1 and the subsequent translocation of rpL10A to the nucleus. Thus, upon geminivirus infection, rpL10A is trapped within the cytoplasm as punctate corpuscles to prevent the establishment of a host environment that disfavors virus proliferation and/or spread.

A question that remains unanswered is how phosphorylated rpL10A functions to impact viral infectivity leading to a delay in the onset of virus infection. Yeast rpL10A is required for joining of the 40S and 60S subunits [33] and for large subunit nuclear export through direct interaction with Nmd3p, a NES (nuclear export signal)-containing protein that is specifically associated with 60S subunits [34]. By analogy with the yeast rpL10A homolog, one may predict that relocation of *Arabidopsis* rpL10A to the nucleus would interfere in both ribosome subunit assembly and 60S subunit export from the nucleus, which would affect general translation and hence impair virus infection. Alternative mechanisms for rpL10A action would invoke extraribosomal functions like those described for other eukaryotic homologs. The *rpL10A* gene (At1g14320) is closely related to QM originally identified from the Wilms' tumor cell line as a candidate tumor-suppressor gene [35] and has been shown to regulate the proto-oncogene *c-Yes* [36]. The chicken QM homolog, designated Jif-1 (Jun interactor factor-1) interacts with the transcriptional factor Jun and influences c*jun*–mediated transcription and apoptosis [23],[37]. Yeast *QM* homologous genes, such as *GRC5* or *QSR1*, participate in translational control of gene expression [38] and an *Entamoeba histolytica QM* homolog exhibits extraribosomal functions associated with suppression of cell proliferation [39]. These putative rpL10A functions, translational control and cell proliferation suppression, may serve as potential host defense strategies against virus. The identification of downstream targets of rpL10A is crucial to decipher this layer of innate defense and to elucidate the underlying mechanism of a defense signaling that has the potential to interfere with normal developmental and cell proliferation events. Because a productive viral infection depends extensively on host biochemical and physiological performance, the NIK-mediated defense responses are likely to target other phytoviruses as well. The NIK1-overexpressing lines will allow us to address the effectiveness of this innate defense strategy on other virus compatible interactions.

In summary, the present characterization of the NIK1-mediated signaling pathway uncovered a novel strategy of plant defenses and provided new insights into the molecular basis for bipartite begomovirus virulence. In fact, this pathway was identified by the virulence function of NSP from CaLCuV that suppresses the NIK1 receptor kinase activity and hence impairs the innate response stoichiometrically. Likewise, the virulence function of NSP from ToYSV is likely to target and inhibit the tomato NIK counterpart, because overexpression of *Arabidopsis NIK1* in tomato delays the onset of geminivirus infection. However, in nonhost-virus interactions, a sustained NIK signaling is likely to prevent other virus from infection in most plants, because NIK is conserved in plant species from the *Leguminosae*, *Solanaceae* and *Brassicaceae* families [8],[9]. Very likely, the NIK-mediated response, which is overcome by geminivirus infection, may be involved in nonhost immunity for other phytoviruses.

## Materials and Methods

A detailed description of methods is provided in Text S1.

### Yeast Two-Hybrid Screen

For the yeast two-hybrid screening, we used a previously described *Arabidopsis thaliana* cDNA library [14],[21] and the pBD-KDNIK1 clone [8]. The screening was performed with the yeast strain MaV203, as described [21].

### Plasmid Construction

The U13033 plasmid harboring a *SERK1* cDNA was obtained from ABRC (*Arabidopsis* Biological Resource Center). The clones pGST-KDNIK1, pGST-KDNIK2, pK7F-NIK1 and pK7F-NIK2 have been described [8]. The clones T474D, 473V/T474A harboring point mutations on the *NIK1* kinase domain sequence (Figure S2, Text S1) and QM104 with point mutation in the rpL10A sequence were obtained through the Gene Tailor Site-directed Mutagenesis system (Invitrogen Life Technologies, Inc.). The plasmids pAD-L10 and pAD-L18 harboring the *rpL10A* cDNA and *rpL18* cDNA, respectively, were isolated by two hybrid screens [21]. All the other recombinant plasmids were obtained through the GATEWAY system (Invitrogen Life Technologies, Inc.). *GST*-fused to *rpL10A*, *rpL18*, *and QM104* or *KDSERK1* sequences were generated by transferring the appropriate DNA fragment from pDONR201 to pDEST15 to yield the clones pGST-L10, pGST-L18, pGST-QM104 and pGST-KDSERK1. Likewise, *rpL10A*, *rpL18*, *NIK1T474D* or *NIK1G473V/T474A* DNAs were transferred from pDONR210 to pK7FWG2 to generate pK7F-L10, pK7F-L18, pK7F-NIK1T474D and pK7F- NIK1G473V/T474A that contain a *GFP* gene fused in-frame after the last codon of the respective cDNAs. To obtain *YFP* gene fused before the first codon of *rpL10A* or *rpL18* genes, the respective cDNAs were transferred from pDONR207 to 35S-YFP-casseteA-Nos-pCAMBIA1300, yielding pYFP-L10 and pYFP-L18.

### Purification of GST-Fusion Proteins

The plasmids pGST-L10, pGST-L18, pGST-QM104, pGST-KDNIK1, pGST-KDNIK2, pGST-KDSERK1, pGST-KDNIK1T474D and pGST-KDNIK1G473V/T474A were transformed into *E. coli* strain BL21, and the synthesis of the recombinant protein was induced by 0.4 mM isopropyl-β-D-thiogalactopyranoside for 16 h at 22°C. The GST fusions were affinity-purified using GST-Sepharose beads (Qiagen), according to manufacturer's instructions.

### Protein Kinase Assay

Purified GST-KDNIK1, GST-KDNIK2, GST-KDNIK3, GST-KDNIK1T474D or GST-KDNIK1G473V/T474A fusion proteins were incubated alone or with GST-L10, GST-QM104 or GST-L18 for 45 min at 25°C in 30 µL of kinase buffer containing 18 mM HEPES (pH 7.4), 10 mM MgCl2, 10 mM MnS04, 1 mM DTT, 10 µM ATP, and 5 µCi [γ-^32^P]ATP. As for GST-KDSERK1, phosphorylation reactions were performed as described [40]. Phosphoproteins were resolved by SDS-PAGE. The gel was stained with coomassie brilliant blue to verify protein loading, dried, and subjected to autoradiography. Incorporated radioactivity in protein bands was quantified by phosphoimaging and protein loading by densitometry using the Multi Gauge V3.0 software (Fujifilm).

### Subcellular Localization of Proteins

*Nicotiana tabacum* leaves were agroinoculated with pK7F-L10, pYFP-L10, pK7F-L18, pYFP-L18, pK7F-NIK1, pK7FNIK2, pK7F-NIK3, pK7F-NIK1T474D or pK7F-NIK1G473V/T474A using *Agrobacterium tumefaciens* strain GV3101, as described [13],[41]. About 72 hours postagroinfiltration, 1-cm^2^ leaf explants were excised and GFP and YFP fluorescence patterns were examined in epidermal cells by confocal microscopy (see Text S1).

### Immunoblotting of Nuclear Extracts

Tobacco leaves were agroinoculated with pYFP-L10, pK7F-NIK1, pK7F-NIK1T474D or pK7F-NIK1G473V/T474A. Nuclear extracts were prepared from agroinfiltrated tobacco leaves as previously described [42], separated by SDS-PAGE and immunoblotted with anti-GFP antiserum (Text S1).

### Protoplast Isolation and Co-Immunoprecipitation of Ectopically Expressed Proteins

Protoplasts were prepared from leaves of 4 to 6 week old tobacco plants that had been agroinfiltrated with the constructions as indicated in the figure. Frozen protoplasts were homogenized with two volumes of ice-cold buffer (150 mM Tris/HCl, 150 mM NaCl, 1.5 mM EDTA and 1.5% (v/v) Triton X-100, pH 7.5) supplemented with 0.1 mM PMSP. Cell homogenates from 2×10^6^ cells from leaf protoplasts expressing NIG or NSP-YFP or both proteins were incubated with rabbit polyclonal antisera raised against GFP (Invitrogen) and protein A-Sepharose. Immunoselected proteins were analyzed by SDS-PAGE and blotted onto nitrocellulose membranes. The membranes were blocked in NaCl-Tris containing 0.05% (v/v) Tween 20 and 1% (w/v) nonfat dry milk, and then incubated with a rabbit anti-GFP or rabbit anti-rpL10A serum for 2 h at room temperature. Bound antibody was detected using an alkaline phosphatase-conjugated goat anti-rabbit IgG serum in conjunction with nitroblue tetrazolium/5-bromo-4-chloro-3-indolyl phosphate (Bio-Rad, UK) detection reagents.

### Plant Material, Growth Conditions, and Genotyping

The *rpL10A* and *nik1* mutants were from the SALK Institute (SALK_010170). Plants were grown in a growth chamber at 22°C under long-day conditions (16 h light/8 h dark). The genotyping of SALK_010170 seeds was performed by PCR and gene inactivation confirmed by RT-PCR (Text S1).

### Tomato Transformation

Tomato leaf discs (*Solanum lycopersicum*, cultivar Moneymaker) were transformed with pK7-NIK1 harboring *AtNIK1* cDNA under control of 35S promoter [8] via Agrobacterium-mediated plant transformation (Text S1). Regenerated shoots were rooted, transferred into soil, and grown in standardized greenhouse conditions (T0 plants) to generate seeds. For infectivity assays, we used T1 transgenic plants harboring the *NIK1* gene construct, which were derived from two independently regenerated kanamycin-resistant plants (35S-NIIK1-4 and 35S-NIK1-6). Analysis of transgene expression was performed by RT-PCR with transgene-specific primers, as described [43]. In control reactions, we used endogenous *NIK* homolog (SlNIK) gene-specific primers for the RT-PCR assays.

### CaLCuV and ToYSV Inoculation and Analysis of Infected Plants

*Arabidopsis thaliana* plants at the seven-leaf stage were inoculated with plasmids containing partial tandem repeats of CaLCuV DNA-A and DNA-B by biolistic delivery and the course of infection was monitored as described [8],[14],[21]. Tobacco leaves were biolistically inoculated with partial tandem repeats of *Tomato golden mosaic virus* (TGMV) DNA-A and DNA-B [26]. Likewise, NIK1-overexpressing tomato plants at the six-leaf stage were inoculated with tandem repeats of ToYSV DNA-A and DNA-B [29] by biolistic delivery [44]. Total nucleic acid was extracted from systemically infected leaves, and viral DNA was detected by PCR with DNA-A or DNA-B specific primers.

### RNA Silencing Assay

*A. tumefaciens* cultures harboring DNA constructs expressing GFP (silencing target), dsGFP (the silencing inducer), HC-Pro (a silencing suppressor), rpL10A or NIK1 were mixed in the combinations indicated in the figure legend and infiltrated into *Nicotiana benthamiana* leaves. Inactivation of the endogenous *NbL10* gene was induced with dsL10-expressing construct, since *AtL10* and *NbL10* are highly conserved. GFP accumulation was visualized under UV light and RNA expression monitored by RT-PCR (Text S1).

### Real-Time RT-PCR Analysis

Real-time RT-PCR reactions were performed as previously described [45]. For quantitation of gene expression in tobacco leaves, we used actin as the endogenous control gene.

## Supporting Information

Text S1 Supplemental Methods. Detailed description of materials and methods.
(0.10 MB PDF)

Figure S1 The cytosolic rpL10 serves as a specific substrate for NIK1. (A) In vitro phosphorylation assays using rpL10 and rpL18 as substrates of LRRI-RLK members. Bacterially produced GST-fusion proteins (as indicated) were purified, and aliquots of 200–500 ng were incubated with [γ^32^P]ATP in the presence of rpL10 or rpL18. After separation on 10% SDS-PAGE, the phosphoproteins were visualized by autoradiography. (B) Confocal fluorescence images of epidermal cells of tobacco leaves agroinoculated with GFP or YFP under the control of the 35S promoter. Scale bars are 10 µm. (C) Cytosolic and nuclear localization of rpL10. Tobacco leaves were agroinoculated with YFP-rpL10 or rpL10-GFP, and images were taken by confocal laser scanning microscopy 72 hours post-transfection. Full arrows indicate fluorescent nuclei observed in a small fraction of transfected cells. Note: In mature leaf epidermal cells, due to the large central vacuole, the cytoplasm is pushed up against the plasma membrane and appears as a narrow area in confocal slices. (D) Subcellular localization of rpL18. Full arrows indicate fluorescent nucleoli.
(3.82 MB TIF)

Figure S2 Schematic representation of mutations in NIK1 and rpL10 sequences. (A) Sequence alignment of the activation segment among NIKs and SERK1. The activation segment is a region of the protein kinases that has been shown to regulate kinase function. The conserved secondary elements in this segment are the magnesium binding loop, β9, at the N-terminus, the centrally located activation loop, and the P+1 loop at the C-terminus. The activation segment of NIK1 was aligned to its counterpart from tomato (SlNIK) and from soybean (GmNIK) and to SERK1 using the ClustalW program. The arrow indicates the conserved threonine residue within the A-loop that has been shown to be essential for kinase activation. T474A and G473V/T474A indicate the mutations (in red) within the NIK1 A-loop. (B) Sequence alignment of a conserved region of rpL10 proteins. The corresponding regions of rpL10s from *E.coli* (83287893), *T. Thermophilus* (58177182), *H. marismortui* (55379031), *S. cereviseae* (6323104), *H. sapiens* (131762), *S. lycopersicum* (AAY97865), and *A. thaliana* (30683726) were compared using the ClustalW program. Arrow indicates the serine residue that was mutated to alanine in the rpL10 from *Arabidopsis* to give the mutant QM104 that is defective for NIK-mediated phosphorylation. (C) SDS-PAGE of *E. coli*-produced GST fusions. GST fused to the C-terminal kinase domain of normal NIK (GST-KDNIK) or to mutant NIK1s (T474D and G473V/T474A) as well as to rpL10 or mutant rpL10 (QM104) were produced in *E. coli*, affinity-purified, separated by SDS/PAGE, and stained with coomassie brilliant blue. Molecular mass markers (kDa) are shown on the left.
(4.41 MB TIF)

Figure S3 Accumulation of rpL10 and mutant QM104 in transfected epidermal leaf cells. Protein extracts from protoplasts prepared from rpL10-GFP–transfected leaves (lane L10), as well as from leaves co-transfected with rpL10-GFP and NIK1 (lane NIK + L10) or with QM104-GFP and NIK1 (lane NIK + QM104) were immoprecipitated with an anti-GFP serum, separated by SDS-PAGE and immunoblotted using a GFP antibody.
(0.11 MB TIF)

Figure S4 Identification of *rpl10* mutant alleles. (A) Annotated rpL10 genomic loci and diagram of the T-DNA insertion. The gene is indicated in the 5′–3′ orientation. Black boxes represent the exons. The position of T-DNA insertion in the null allele is indicated. (B) Homozygous population of T-DNA insertional *rpl10* mutants. Total DNA was extracted from leaves of rpl10 progenies (l10-KO), and T-DNA insertion in *rpL10* locus was monitored by PCR. M corresponds to DNA standard markers, and Col-0 is Columbia. (C) Analysis of *rpL10* transcripts. RT-PCR was performed on leaf RNA samples from wild-type (Col-0) and rpl10 plants with gene-specific primers. The positions of DNA standard markers are shown on the left in kbp.
(0.34 MB TIF)

Figure S5 TGMV infection of *Nicotiana tabacum* leaves. (A) Symptomatic tobacco leaves. Tandemly repeated TGMV DNA-A and DNA-B were introduced into tobacco plants by biolistic inoculation. On the top, the indicated plants were bombarded with tungsten particle without viral DNA. The bottom shows infected plants at 7 days postinoculation (DPI). (B)Viral DNA accumulation in infected lines. Total DNA was extracted from inoculated leaves, and viral DNA was detected with DNA-B–specific primers. IN refers to TGMV-inoculated plants and UN to mock-inoculated plants. + indicates control plasmid DNA as template. (C) Subcellular localization of CaLCuV NSP in infected plants. Both uninfected (top) and infected (bottom) tobacco leaves were agroinfiltrated with YFP-NSP, and the subcellular localization of the YFG-tagged protein was visualized by confocal microscopy.
(1.63 MB TIF)

Figure S6 Titration of NSP inhibitor. Increasing amounts of GST-KDNIK1 were incubated with [γ-^32^P]ATP in the presence of GST (30 ng/µL) or GST-NSP (60 ng/µL). After separation on SDS-PAGE, phosphoproteins were visualized by autoradiography and quantified by phosphoimaging. Relative values of ^32^P incorporation are the mean of three replicas.
(0.45 MB TIF)

Figure S7 rpL10 is not involved in RNA silencing mechanisms. *N. benthamiana* leaf tissues were coinfiltrated with Agrobacterium tumefaciens carrying GFP-, dsGFP-, indicated cDNAs-, or inverted repeat RNA-expressing constructs. (A) Photographies were taken under UV lights 5 days postinfiltration. (B) Transcript accumulation in infiltrated leaves. The accumulation of the indicated transcripts was determined by semi-quantitative RT-PCR on RNA extracted from infiltration zones 5 days postinfiltration with gene-specific primers. GAP was used as control.
(3.70 MB TIF)

Figure S8 NIK-mediated defense signaling does not induce a hypersensitive response. Tobacco leaf tissues were coinfiltrated with Agrobacterium cultures delivering Ti plasmids expressing rpL10, rpL10 + NIK1, rpL10 + inactive mutant NIK1 (mNIK1), rpL10 +hyperactive mutant NIK1 (T474D), or a control hypersensitive response-inducing soybean NAC6 protein, as positive control, and, as negative control, the NSP-interacting GTPase from *Arabidopsis*. (A) Leaf necrotic symptoms typical of inducers of hypersensitive response. Leaf sections were agroinfiltrated with the indicated agroinoculum, and pictures were taken 6 days after infiltration. (B) Expression of pathogenesis-related (PR) genes in agroinfiltrated leaves. Two days postinfiltration with the indicated agroinoculum, RNA was extracted from the infiltration zones, and expression of the pathogenesis-related genes PR-1, PR-4, and chitinase was analyzed by RT-PCR. Values are relative to control treatment and represent the mean±SD of three replicates from three independent experiments.
(4.72 MB TIF)
