# Supplementary material for: Regulated Nuclear Trafficking of rpL10A Mediated by NIK1 Represents a Defense Strategy of Plant Cells against Virus
Source: PLoS Pathog. 2008 Dec 26;4(12):e1000247. doi: 10.1371/journal.ppat.1000247 (PMC2597721; doi:10.1371/journal.ppat.1000247)
Supplement: Text S1 — Supplemental Methods. Detailed description of materials and methods. (0.10 MB PDF) [file ppat.1000247.s001.pdf]

Text S1- Supplemental Methods

Plasmid construction. The U13033 plasmid harboring a *SERK1* cDNA was obtained from ABRC (Arabidopsis Biological Resource Center). pGST-KDNIK1 and pGST-KDNIK2 containing the kinase domain-encoding DNA of *NIK1* or *NIK2* fused to *GST*, as well as pK7F-NIK1 and pK7F-NIK2 containing *NIK1* or *NIK2* cDNA fused to *GFP* have been previously described [8]. The point mutation T474D and the double mutations G473V/T474A within the activation loop (A-loop) of NIK1 kinase domain were generated through the Gene Tailor^{TM} Site-directed Mutagenesis system (Invitrogen Life Technologies, Inc, Figure S2A). The clones pGST-KDNIK1T474D and pGST-KDNIK1G473V/T474A harbor point mutations on the *NIK1* kinase domain sequence fused to *GST* and pK7F-NIK1G473VT474A clone contains the double mutant *NIK1* sequence fused to *GFP*. The plasmids pAD-L10 and pAD-L18 harboring the *rpL10* cDNA and *rpL18* cDNA, respectively, were isolated by two hybrid screens. The clone pQM104 in which a point mutation changed a serine residue, position 104, to an alanine residue were obtained through the Gene Tailor^{TM} Site-directed Mutagenesis system (Invitrogen Life Technologies, Inc, Figure S2B) and then transferred from pDONR201 to pDEST15 to generate pGST-QM104. All the other recombinant plasmids were obtained through the GATEWAY system (Invitrogen Life Technologies, Inc.). Briefly, the specified DNA fragments were amplified by PCR with appropriate extensions and introduced by recombination into the entry vector pDONR201 or pDONR207 and then transferred to the appropriate destination vector. A description of the recombinant plasmids is provided below.

GST-fused to *rpL10, rpL18* or *KDSERK1* sequences were generated by transferring the appropriate DNA fragment from pDONR201 to the bacterial expression vector pDEST15 to yield the clones pGST-L10, pGST-L18 and pGST-KDSERK1. Likewise, *rpL10*, *rpL18*, *NIK1T474D* or *NIK1G473V/T474A* DNAs were transferred from pDONR210 to the binary

vector pK7FWG2 to generate pK7F-L10, pK7F-L18, pK7F-NIK1T474D and pK7F-NIK1G473V/T474A that contain a *GFP* gene fused in-frame after the last codon of the respective cDNAs. To obtain *YFP* gene fused before the first codon of *rpL10* or *rpL18* genes, the respective cDNAs were transferred from pDONR207 to 35S-YFP-casseteA-Nos-pCAMBIA1300 (gift from Dr Steve Slocombe), yielding pYFP-L10 and pYFP-L18. rpL10 cDNA was also transferred from pDONR201 to pK7GWiG to generate dsL10, which contains an inverted rpL10 cDNA under control of 35S promoter.

Purification of GST- fusion proteins. The expression plasmids pGST-L10, pGST-L18, pGST-KDNIK1, pGST-KDNIK2, pGST-KDSERK1, pGST-KDNIK1T474D and pGST-KDNIK1G473V/T474A were transformed into *E. coli* strain BL21, and the synthesis of the recombinant protein was induced by 0.4 mM isopropyl-β-D-thiogalactopyranoside for 16 h at 22°C. Accumulation of recombinant protein was monitored by SDS–PAGE in whole cell extracts, as well as in soluble and insoluble fractions. Cells were pelleted by centrifugation, resuspended in lysis buffer, disrupted by sonication, and centrifuged at 39,000g for 20 min. The GST fusions were affinity-purified using GST-Sepharose beads (Qiagen), according to manufacturer’s instructions. The efficiency of protein purification was monitored by SDS-PAGE.

Protein kinase assay. Purified GST-KDNIK1, GST-KDNIK2, GST-KDNIK3, GST-KDNIK1T474D or GST-KDNIK1G473V/T474A fusion proteins were incubated alone or with GST-L10, GST-QM104 or GST-L18 for 45 min at 25°C in 30 μL of kinase buffer containing 18 mM HEPES (pH 7.4), 10 mM MgCl2, 10 mM MnS04, 1 mM DTT, 10 μM ATP, and 5 μCi [γ-^{32}P]ATP. As for GST-KDSERK1, phosphorylation reactions were performed as described [40]. Phosphoproteins were resolved by SDS-PAGE. The gel was stained with Coomassie brilliant blue to verify protein loading, dried, and subjected to autoradiography.

Incorporated radioactivity in protein bands was quantified by phosphoimaging and protein loading by densitometry using the Multi Gauge V3.0 software (Fujifilm).

Subcellular localization of proteins. For subcellular localization of proteins, *Nicotiana tabacum* leaves were agroinoculated with pK7F-L10, pYFP-L10, pK7F-L18, pYFP-L18, pK7F-NIK1, pK7FNIK2, pK7F-NIK3, pK7F-NIK1T474D or pK7F-NIK1G473V/T474A using *Agrobacterium tumefaciens* strain GV3101. *Nicotiana tabacum* plants were grown in a greenhouse with natural day length illumination. For experimental use, plants (about three week after germination) were transferred to a growth chamber at 21°C with a 16-hour light and 8-hour dark cycle. Each construct was mobilized in *Agrobacterium tumefaciens* strain GV3101 by freeze-thawing followed by selection on YEB plates containing the appropriate antibiotics. Agrobacterium-mediated transient expression in tobacco leaf epidermal cells was conducted as previously described [13, 41]. About 72 hours post-agroinfiltration, 1-cm^2 leaf explants were excised and GFP and YFP fluorescence patterns were examined in epidermal cells with 40X or 60X oil immersion objective and a Zeiss inverted LSM510 META laser scanning microscope equipped with an argon laser and a helium laser as excitation source. For imaging GFP, the 458-488 nm excitation line and the 500 to 530 nm band pass filter were used. Excitation of YFP was at 514-560 nm and YFP emission was detected by using a 560-600 nm filter. Controls were performed to ensure clear separation of GFP and YFP signals. The pinhole was usually set to give a 1 to 1.5 μm optical slice. Post-acquisition image processing was done using the LSM 5 Browser software (Carl-Zeiss) and Adobe Photoshop (Adobe Systems).

Immunoblotting of nuclear extracts. Tobacco leaves were agroinoculated with pYFP-L10, pK7F-NIK1, pK7F-NIK1T474D or pK7F-NIK1G473V/T474A using *Agrobacterium tumefaciens* strain GV3101. Nuclear extracts were prepared from agroinfiltrated tobacco

leaves as previously described [42]. The integrity of the nuclear extracts was monitored by SDS-PAGE. Equivalent amounts of nuclear protein (30 μg) were resolved by SDS-PAGE on a 15% polyacrylamide gel and transferred to nitrocellulose membranes by electroblotting. The membrane was blocked with 3% (w/v) bovine serum albumin in TBST [100 mm TRIS-HCl (pH 8), 150 mm NaCl, 0.05% (v/v) Tween 20]. YGP-L10 was detected using policlonal anti-GFP antibody (Invitrogen) at a 1:10000 dilution, followed by a goat anti rabbit IgG conjugated to alkaline phosphatase (Sigma) at a 1:5000 dilution. The activity of alkaline phosphatase was assayed using 5-bromo-4- chloro-3-indolyl phosphate and p-nitroblue tetrazolium (Life Technologies). For the dephosphorylation assays, nuclear protein extracts were treated with 20 units of alkaline phosphatase for 2 h at 37 °C prior to electrophoresis.

Protoplast isolation and co-immunoprecipitation of ectopically expressed proteins. Protoplasts were prepared from leaves of 4 to 6 week old tobacco plants that had been agroinfiltrated with the constructions as indicated in the Figure 2, as described [13]. Frozen protoplasts were homogenized with two volumes of ice-cold buffer (150 mM Tris/HCl, 150 mM NaCl, 1.5 mM EDTA and 1.5% (v/v) Triton X-100, pH 7.5) supplemented with 0.1 mM PMSP. Cell homogenates from 2 x 10^6 cells from leaf protoplasts expressing NIG or NSP-YFP or both proteins were incubated with rabbit polyclonal antisera raised against GFP (Invitrogen) and protein A-sepharose. Immunoselected proteins were analyzed by SDS-PAGE and blotted onto nitrocellulose membranes. The membranes were blocked in NaCl-Tris containing 0.05% (v/v) Tween 20 and 1% (w/v) nonfat dry milk, and then incubated with a rabbit anti-GFP or rabbit anti-rpL10 serum for 2 h at room temperature. Bound antibody was detected using an alkaline phosphatase-conjugated goat anti-rabbit IgG serum in conjunction with nitroblue tetrazolium/5-bromo-4-chloro-3-indolyl phosphate (Bio-Rad, UK) detection reagents.

Plant material, growth conditions, and genotyping. The Columbia (Col-0) ecotype of Arabidopsis thaliana was used as the wild type. The *rpl10* and *nik1* mutants were from the SALK Institute (SALK_010170). Seeds were surface sterilized and cold treated at 4°C for 2 days in the dark and then exposed to white light. Seedlings were grown at 22°C on plates containing Murashige-Skoog medium for 3 weeks and then transferred to soil. Plants were grown in a growth chamber at 22°C under long-day conditions (16 h light/8 h dark). The genotyping of SALK_010170 seeds was performed by PCR. Wild-type rpL10 alleles were identified with the rpL10-Fwd (GAACTCTCAGCCTTGCATGAC) and rpL10-Rvs (TCATGGGAGAAGGAGAATGTG) primers and, for detection of the *rpl10* alleles, the rpL10-Fwd primer was used in combination with the SALK LBa1 (TGGTTCACGTAGTGGGCCATCG) primer.

Reverse transcription (RT)-PCR. Total RNA was extracted from the plant tissues using TRIzol (Invitrogen). Reverse transcription (RT)-PCR assays were performed with gene-specific primers for the cDNAs indicated in the figures. The presence of contaminating DNA was assessed in control reactions conducted without reverse transcriptase. The PCR comprised 40 cycles of 45 s at 94°C, 1 min and 30 sec at 50°C, and 1.min at 72°C. When *Nicotiana benthamiana* cDNA was used, PCR was also carried out with GAP-specific primers to assess the quantity and quality of the cDNA.

Tomato transformation. Leaf discs from *in vitro*-grown tomato (*Solanum lycopersicum*, cultivar Moneymaker) plants were co-cultivated for 15 min with *Agrobacterium tumefaciens* strain LBA4404 containing the binary plasmid pK7-NIK1 that harbors the *Arabidopsis NIK1* cDNA under control of 35S promoter [8]. Transformed shoots were selected on MS medium supplemented with 6-benzylaminopurine (500 mg.L-1), cefotaxime (300 mg.L-1), and kanamycin sulfate (50 mg.L-1). Regenerated shoots were rooted, transferred into soil, and

grown in standardized greenhouse conditions (T0 plants) to generate seeds. For infectivity assays, we used T1 transgenic plants harboring the *NIK1* gene construct, which were derived from two independently regenerated kanamycin-resistant plants (35S-NIIK1-4 and 35S-NIK1-6). Analysis of transgene expression was performed by RT-PCR with transgene-specific primers, as described [43]. In control reactions, we used endogenous *NIK* homolog (SlNIK) gene-specific primers for the RT-PCR assays.

Begomovirus inoculation and analysis of infected plants. *Arabidopsis thaliana* plants at the seven-leaf stage were inoculated with plasmids containing partial tandem repeats of CaLCuV DNA-A and DNA-B by biolistic delivery and the course of infection was monitored as described previously [8, 14, 21]. We used attenuated forms of the virus, in which the coat protein ORF in CaLCuV DNA-A was interrupted by introducing a stop codon at amino acid position 47. Total nucleic acid was extracted from systemically infected leaves, and viral DNA was detected by PCR with DNA-A or DNA-B specific primers. In each experiment, 20 plants of each line (Col-0, *nik1* and *rpl10* null alleles) were inoculated with 2 µg of tandemly repeated DNA-A plus DNA-B per plant and grown in a growth chamber at 22°C under long-day conditions (16 h light/8 h dark). The course of infection was registered with data from three independent experiments. $DP1^{50\%}$ (days post- inoculation to reach 50% of infected plants) was obtained with data from five independent experiments. Untransformed and AtNIK1-overexpressing tomato plants at the six-leaf stage were inoculated with plasmids containing partial tandem repeats of ToYSV DNA-A and DNA-B [29] by biolistic delivery as described (44). Inoculated tomato plants were grown in greenhouse conditions (avg. 21°C, max. 31°C, min. 15°C) under natural conditions of light, relative humidity 70%, and approximately equal day and night length. Symptoms were recorded at regular intervals and ToYMV infection was confirmed by PCR with DNA-A or DNA-B degenerate primers. In each experiment, 20 plants of each line (wild type, 35S-NIK1-4 and 35S-NIK1-6) were inoculated with 2 µg of tandemly

repeated DNA-A plus DNA-B per plant. The course of infection was registered with data from three independent experiments.

RNA silencing assay. *Nicotiana benthamiana* plants were grown in a growth chamber at 21°C with a 16-hour light and 8-hour dark cycle. *Agrobacterium tumefaciens*-mediated transient expression in *Nicotiana benthamiana* plants was performed as described [7]. *A. tumefaciens* cultures (optical density of 600 nm = 1) containing plasmids designed to express GFP, an inverted repeat GFP RNA (dsGFP), L10, an inverted repeat L10 RNA (dsL10), NIK1 or HC-Pro were mixed and coinfiltrated to the underside of leaves using a 3ml syringe. We infiltrated Nicotiana leaves with the dsL10-expressing construct to induce silencing of the endogenous NbL10 gene, since AtL10 and NbL10 are highly conserved. GFP fluorescence was observed using a 100W, longwave UV lamp (Black-Ray Model B 100YP; UV Products). Photographs were taken with a UV filter. The images were processed using Adobe Photoshop. Tissue was harvested from infiltration zones and used for RNA extraction.

Total RNA from cells was extracted using an RNAeasy kit (Qiagen). The RNA was treated with RNase-free DNase (Promega) at 37 °C for 1 h and the DNAse was inactivated at 65 °C for 10 min. Reverse transcription reaction was done using the ImProm-IITm Reverse Transcription System (Promega) according to the manufacturer's instructions. A typical reaction consisted of 1 µl of the reverse transcription reaction, 0.5 mM each dNTP, 100 mM each sense and antisense gene-specific primers, 1 × PCR buffer, 1.5 mM MgCl2), and 1 unit of Taq DNA polymerase (Promega) in a total volume of 20 µl. The primers used are listed in Table S1. The presence of contaminating DNA was assessed in control reactions conducted without reverse transcriptase. The reactions were performed for 25 cycles (30 s at 94 °C, 30 s at 52 °C, and 1 min at 72 °C), with a final extension at 72 °C for 10 min. The reaction products were separated by electrophoresis on 1.5% agarose, Tris borate-EDTA gels and visualized with ethidium bromide-staining under UV transillumination.

Real-time RT-PCR analysis. Real-time RT-PCR reactions were performed as previously described [45] Total RNA was extracted from frozen leaves with TRIzol (Invitrogen) according to the instructions from the manufacturer. The RNA was treated with 2 units of RNase-free DNase (Promega) and further purified through RNeasy Mini Kit (QIAGEN) columns. First-strand cDNA was synthesized from 4 µg of total RNA using oligo-dT (18) and Transcriptase Reverse M-MLV (Invitrogen), according to the manufacturer's instructions.

Real-time RT-PCR reactions were performed on an ABI7500 instrument (Applied Biosystems), using SYBR® Green PCR Master Mix (Applied Biosystems). The amplification reactions were performed as follows: 2 min at 50°C, 10 min at 95°C, and 40 cycles of 94°C for 15 sec and 60°C for 1 min. To confirm quality and primer specificity, we verified the size of amplification products after electrophoresis through a 1.5% agarose gel, and analyzed the Tm (melting temperature) of amplification products in a dissociation curve, performed by the ABI7500 instrument. The primers used are listed in Table S1. For quantitation of gene expression in tobacco leaves, we used RNA actin as the endogenous control gene for data normalization in real-time RT-PCR analysis.

Relative mRNA expression, which is based on the comparison of the target gene expression (normalized to the endogenous control) between experimental and control samples, was quantified using the comparative Ct method: 2^-(ΔCtTreatment – ΔCtControl). Absolute gene expression was quantified using the $2^{-}\Delta CT$ method and values were normalized to the endogenous control.

Table S1. Genes analyzed and primers for qRT-PCR or RT-PCR

Clone description	Clone accession	Forward primer	Reverse primer
AtNIK1[1]	AT5G16000	ATGGGAGCTGCAAGAGGG	ATCTAGGACCAGAGAGCTC
SlNIK1[2]	Ref. 9	AATGCTTGAAGGTGATGG	ACTACAACATTGTCTAAA
AtL10	AT1G14320	ATGGGAAGAAGACCTGCG	GCACCTCTCATACCAGTC
NbL10[3]	CN745823	CGTGGTGTGCCAGATCCAAAG	CCAATAGCAACACGAGCACAG
NbGAP	M14419.1	TGATAGGTTTGGAATTGTGG	AATGCTTGACCTGCTGTCAC
GFP	AB434768.1	ATGAGTAAAGGAGAAGAA	GATGAACTATACAAATAG
PR1	X17680.1	CTGCTAAGGCCGTCGAGATGT	GAACCGAGTTACGCCAAACCA
PR4	X60282.1	AGAGCGCCACAAACGTGAGAT	GCCATGCGAGAGGCTTGTC
Chitinase	X51426.1	GTGGCGTAGGTCCGAATGC	GGGCGAAGTTCCTTTGGTTGTA
NtActin[4]	AB158612.1	AGCAAGGAAATTACCGCATTAGC	ACCTGCTGGAATGTGCTGAGA

[1]At- *Arabidopsis thaliana*
[2]Sl- *Solanum lycopersicum*
[3]Nb- *Nicotiana benthamiana*
[4]Nt- *Nicotiana tabacum*
