# Supplementary figures and images for: Regulated Nuclear Trafficking of rpL10A Mediated by NIK1 Represents a Defense Strategy of Plant Cells against Virus
Source: PLoS Pathog. 2008 Dec 26;4(12):e1000247. doi: 10.1371/journal.ppat.1000247 (PMC2597721; doi:10.1371/journal.ppat.1000247)

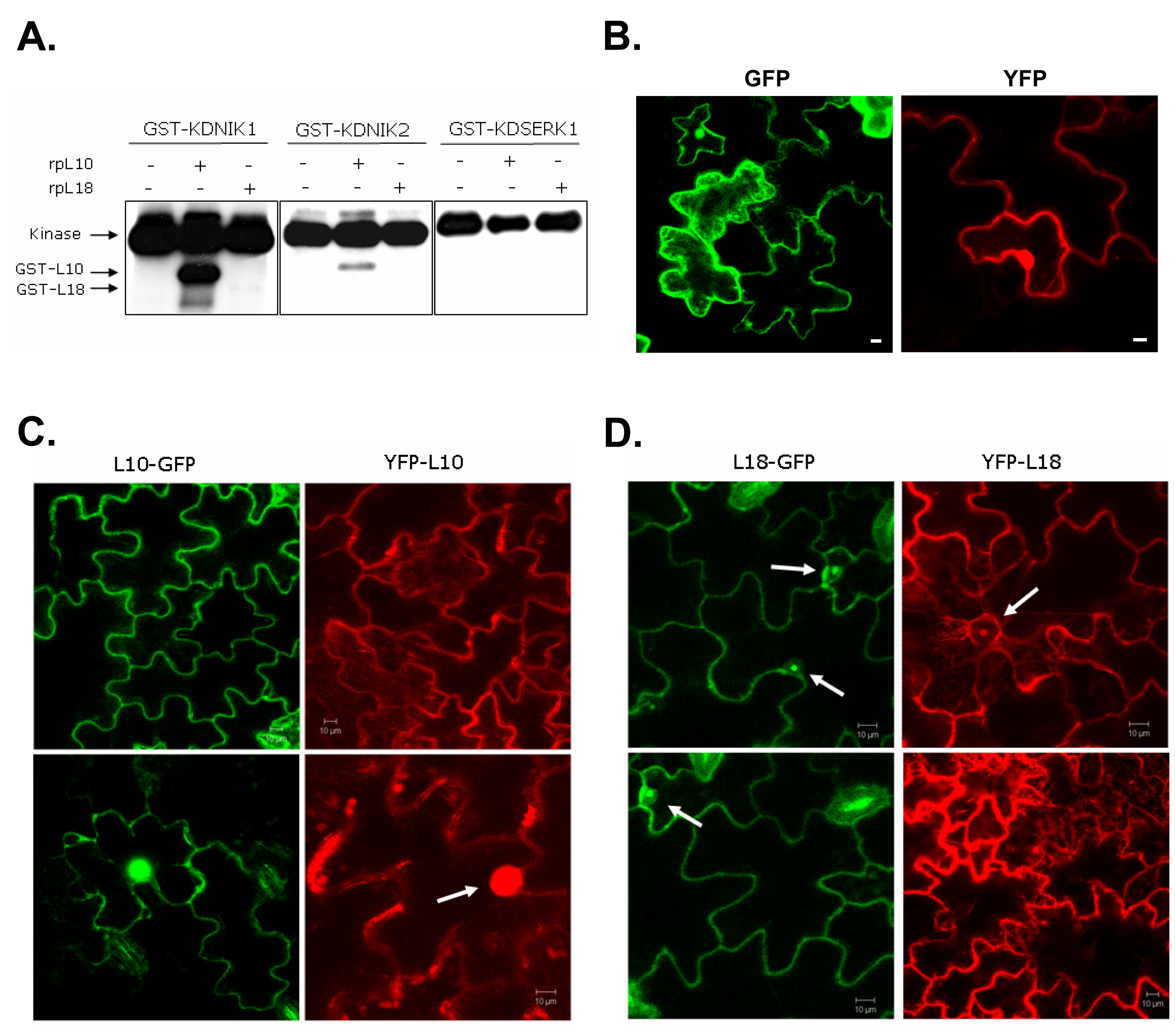

Supplement: Figure S1 — The cytosolic rpL10 serves as a specific substrate for NIK1. (A) In vitro phosphorylation assays using rpL10 and rpL18 as substrates of LRRI-RLK members. Bacterially produced GST-fusion proteins (as indicated) were purified, and aliquots of 200–500 ng were incubated with [γ32P]ATP in the presence of rpL10 or rpL18. After separation on 10% SDS-PAGE, the phosphoproteins were visualized by autoradiography. (B) Confocal fluorescence images of epidermal cells of tobacco leaves agroinoculated with GFP or YFP under the control of the 35S promoter. Scale bars are 10 µm. (C) Cytosolic and nuclear localization of rpL10. Tobacco leaves were agroinoculated with YFP-rpL10 or rpL10-GFP, and images were taken by confocal laser scanning microscopy 72 hours post-transfection. Full arrows indicate fluorescent nuclei observed in a small fraction of transfected cells. Note: In mature leaf epidermal cells, due to the large central vacuole, the cytoplasm is pushed up against the plasma membrane and appears as a narrow area in confocal slices. (D) Subcellular localization of rpL18. Full arrows indicate fluorescent nucleoli. (3.82 MB TIF) [file ppat.1000247.s002.tif]

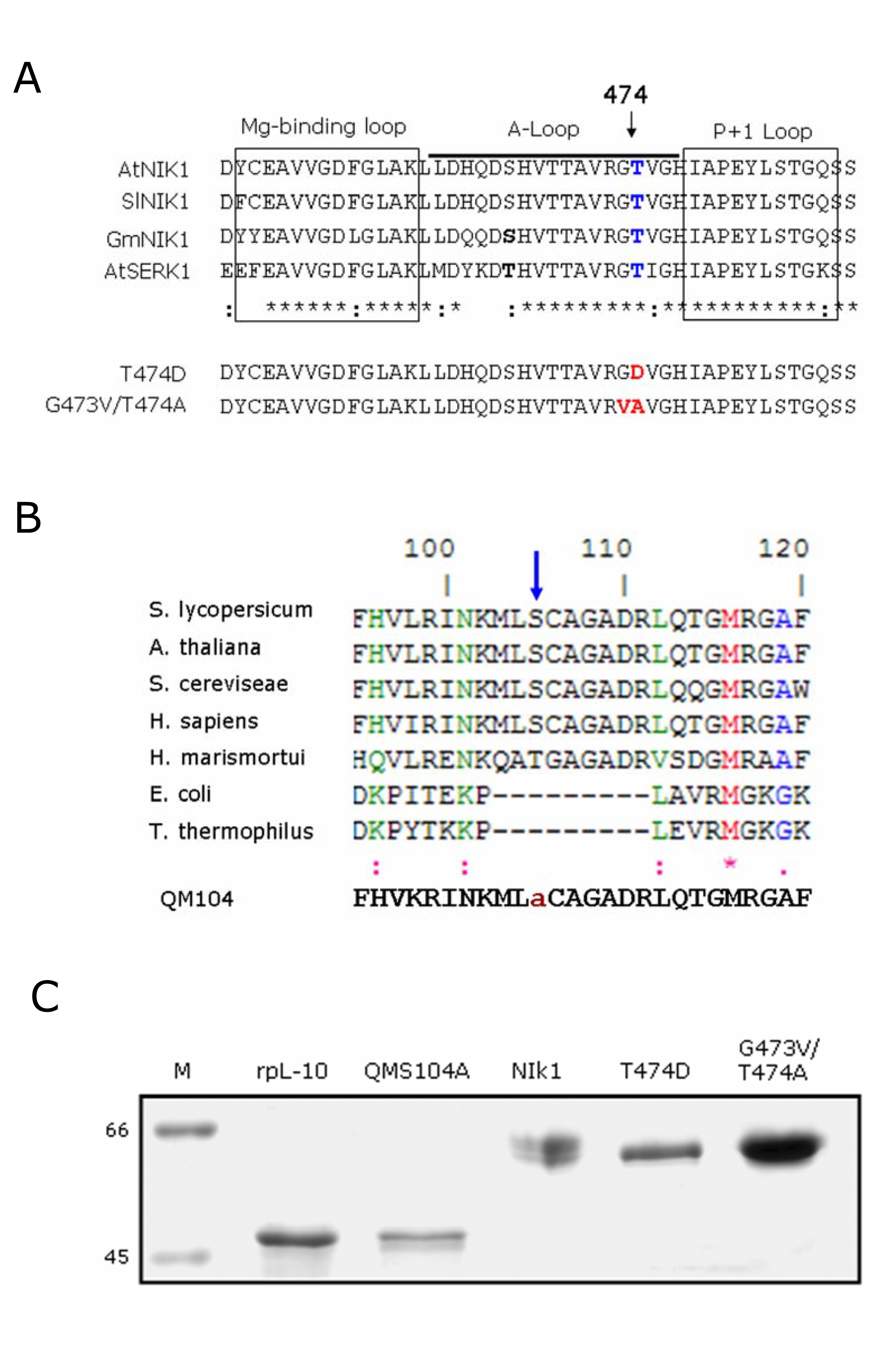

Supplement: Figure S2 — Schematic representation of mutations in NIK1 and rpL10 sequences. (A) Sequence alignment of the activation segment among NIKs and SERK1. The activation segment is a region of the protein kinases that has been shown to regulate kinase function. The conserved secondary elements in this segment are the magnesium binding loop, β9, at the N-terminus, the centrally located activation loop, and the P+1 loop at the C-terminus. The activation segment of NIK1 was aligned to its counterpart from tomato (SlNIK) and from soybean (GmNIK) and to SERK1 using the ClustalW program. The arrow indicates the conserved threonine residue within the A-loop that has been shown to be essential for kinase activation. T474A and G473V/T474A indicate the mutations (in red) within the NIK1 A-loop. (B) Sequence alignment of a conserved region of rpL10 proteins. The corresponding regions of rpL10s from E.coli (83287893), T. Thermophilus (58177182), H. marismortui (55379031), S. cereviseae (6323104), H. sapiens (131762), S. lycopersicum (AAY97865), and A. thaliana (30683726) were compared using the ClustalW program. Arrow indicates the serine residue that was mutated to alanine in the rpL10 from Arabidopsis to give the mutant QM104 that is defective for NIK-mediated phosphorylation. (C) SDS-PAGE of E. coli-produced GST fusions. GST fused to the C-terminal kinase domain of normal NIK (GST-KDNIK) or to mutant NIK1s (T474D and G473V/T474A) as well as to rpL10 or mutant rpL10 (QM104) were produced in E. coli, affinity-purified, separated by SDS/PAGE, and stained with coomassie brilliant blue. Molecular mass markers (kDa) are shown on the left. (4.41 MB TIF) [file ppat.1000247.s003.tif]

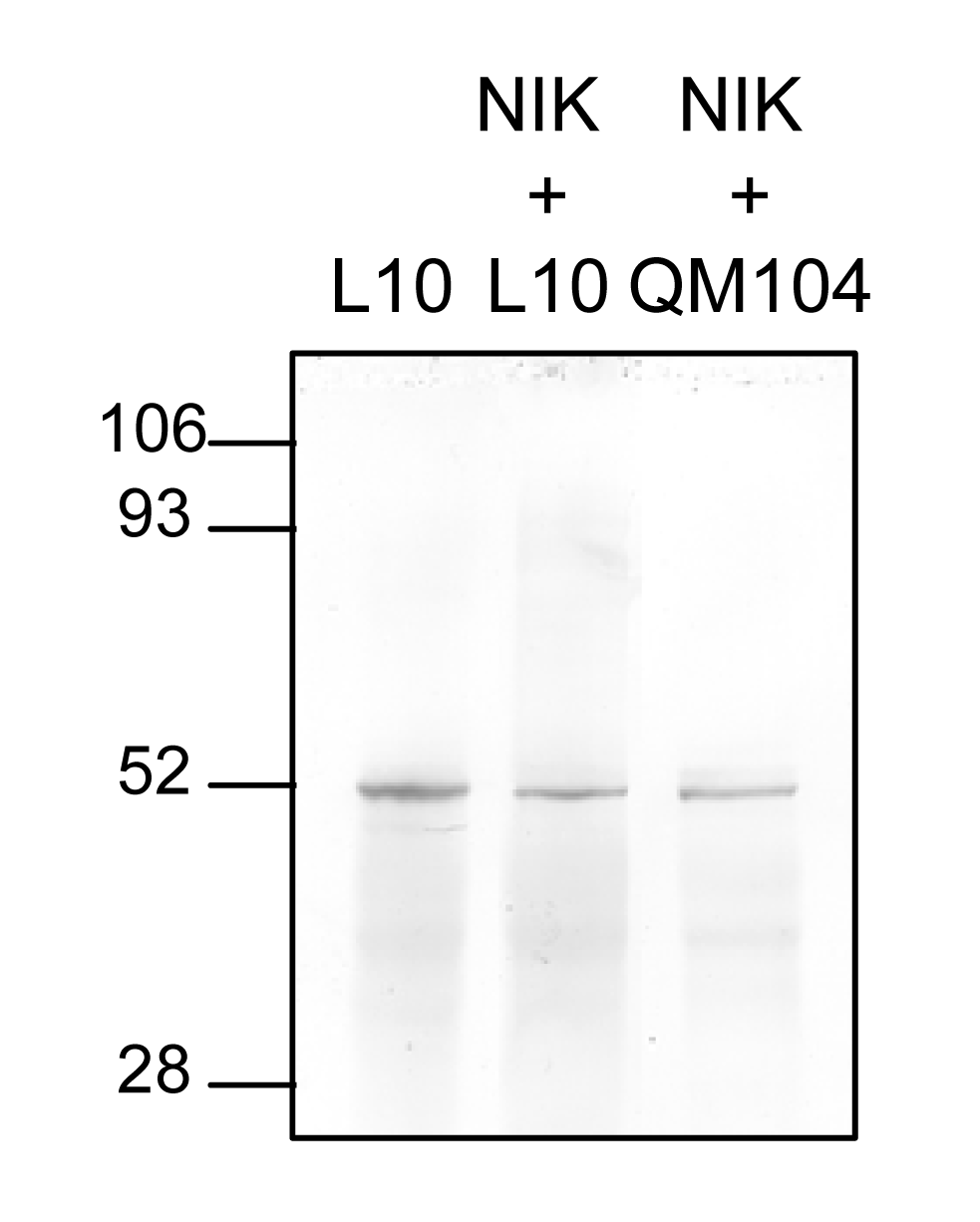

Supplement: Figure S3 — Accumulation of rpL10 and mutant QM104 in transfected epidermal leaf cells. Protein extracts from protoplasts prepared from rpL10-GFP–transfected leaves (lane L10), as well as from leaves co-transfected with rpL10-GFP and NIK1 (lane NIK + L10) or with QM104-GFP and NIK1 (lane NIK + QM104) were immoprecipitated with an anti-GFP serum, separated by SDS-PAGE and immunoblotted using a GFP antibody. (0.11 MB TIF) [file ppat.1000247.s004.tif]

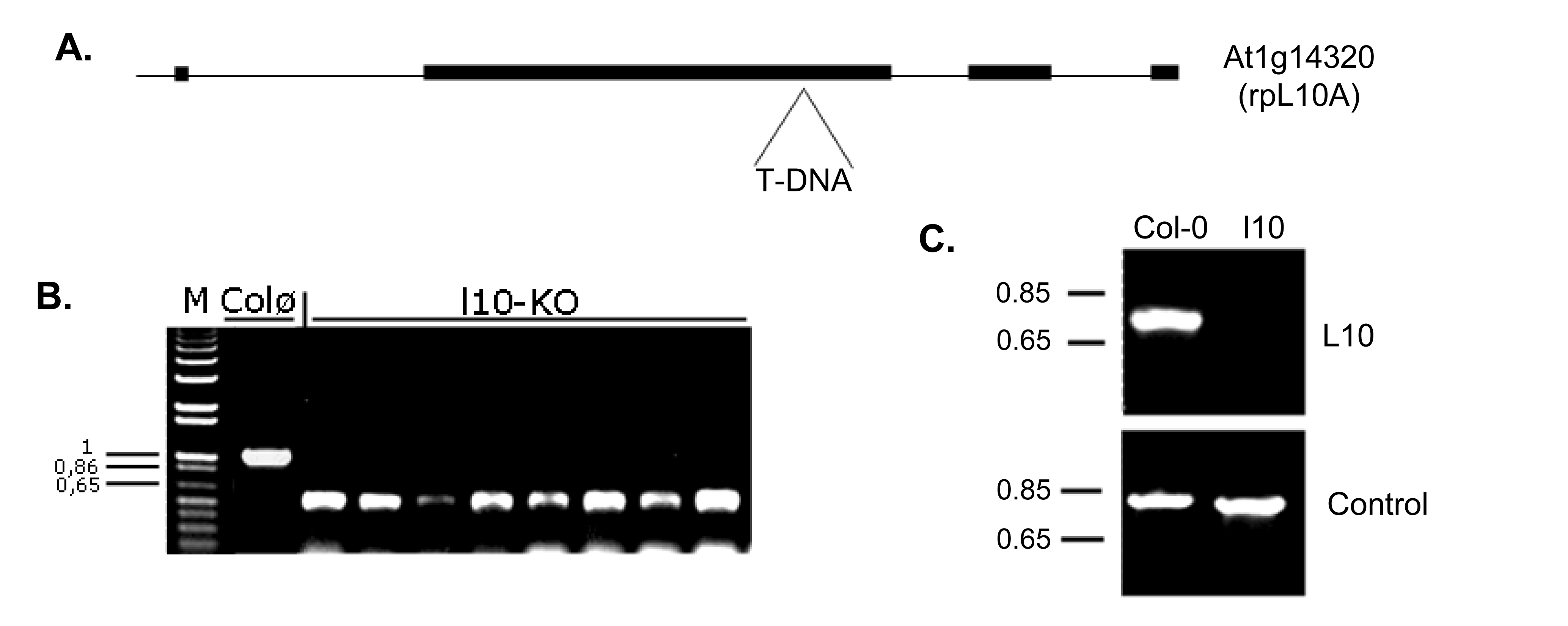

Supplement: Figure S4 — Identification of rpl10 mutant alleles. (A) Annotated rpL10 genomic loci and diagram of the T-DNA insertion. The gene is indicated in the 5′–3′ orientation. Black boxes represent the exons. The position of T-DNA insertion in the null allele is indicated. (B) Homozygous population of T-DNA insertional rpl10 mutants. Total DNA was extracted from leaves of rpl10 progenies (l10-KO), and T-DNA insertion in rpL10 locus was monitored by PCR. M corresponds to DNA standard markers, and Col-0 is Columbia. (C) Analysis of rpL10 transcripts. RT-PCR was performed on leaf RNA samples from wild-type (Col-0) and rpl10 plants with gene-specific primers. The positions of DNA standard markers are shown on the left in kbp. (0.34 MB TIF) [file ppat.1000247.s005.tif]

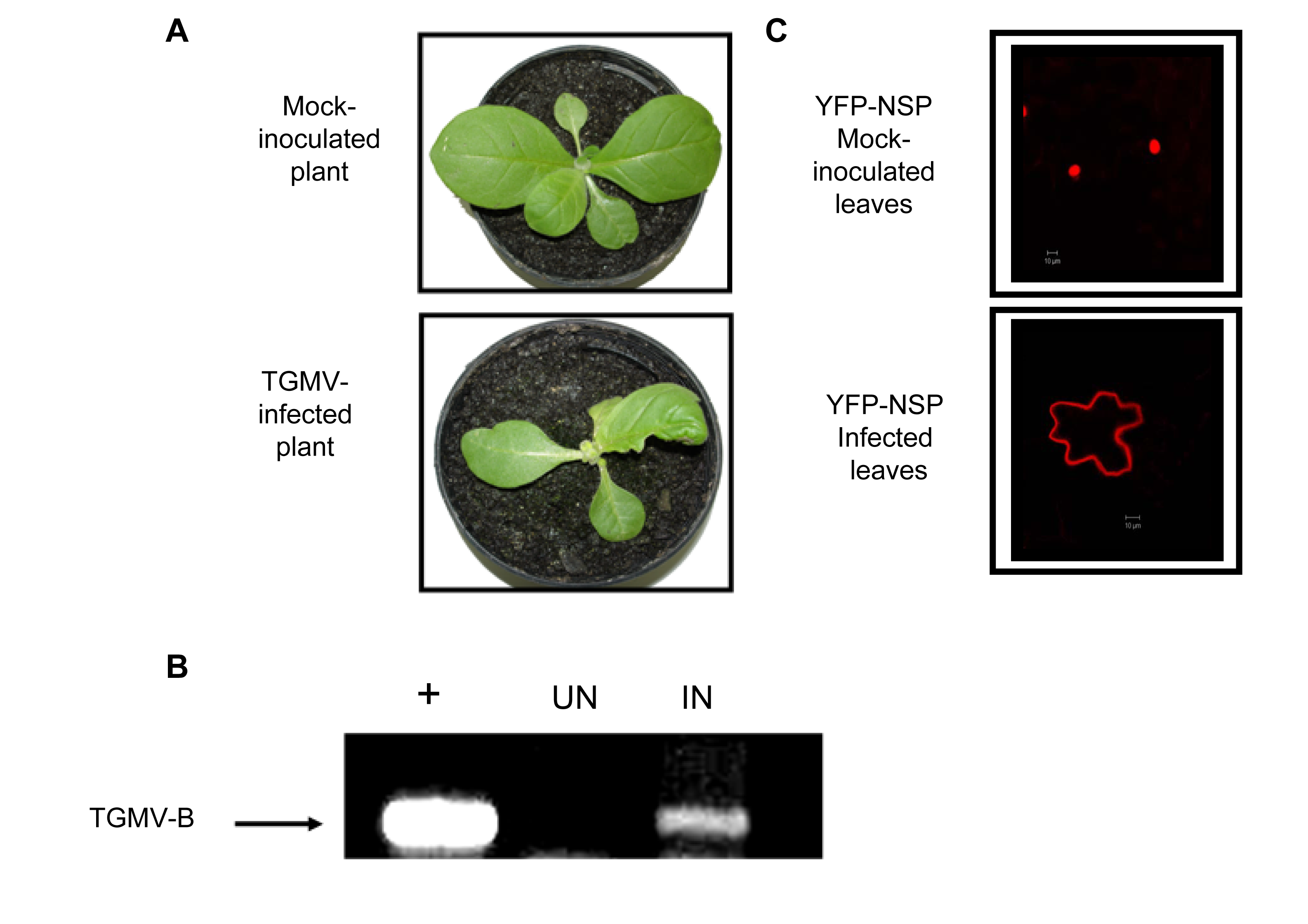

Supplement: Figure S5 — TGMV infection of Nicotiana tabacum leaves. (A) Symptomatic tobacco leaves. Tandemly repeated TGMV DNA-A and DNA-B were introduced into tobacco plants by biolistic inoculation. On the top, the indicated plants were bombarded with tungsten particle without viral DNA. The bottom shows infected plants at 7 days postinoculation (DPI). (B)Viral DNA accumulation in infected lines. Total DNA was extracted from inoculated leaves, and viral DNA was detected with DNA-B–specific primers. IN refers to TGMV-inoculated plants and UN to mock-inoculated plants. + indicates control plasmid DNA as template. (C) Subcellular localization of CaLCuV NSP in infected plants. Both uninfected (top) and infected (bottom) tobacco leaves were agroinfiltrated with YFP-NSP, and the subcellular localization of the YFG-tagged protein was visualized by confocal microscopy. (1.63 MB TIF) [file ppat.1000247.s006.tif]

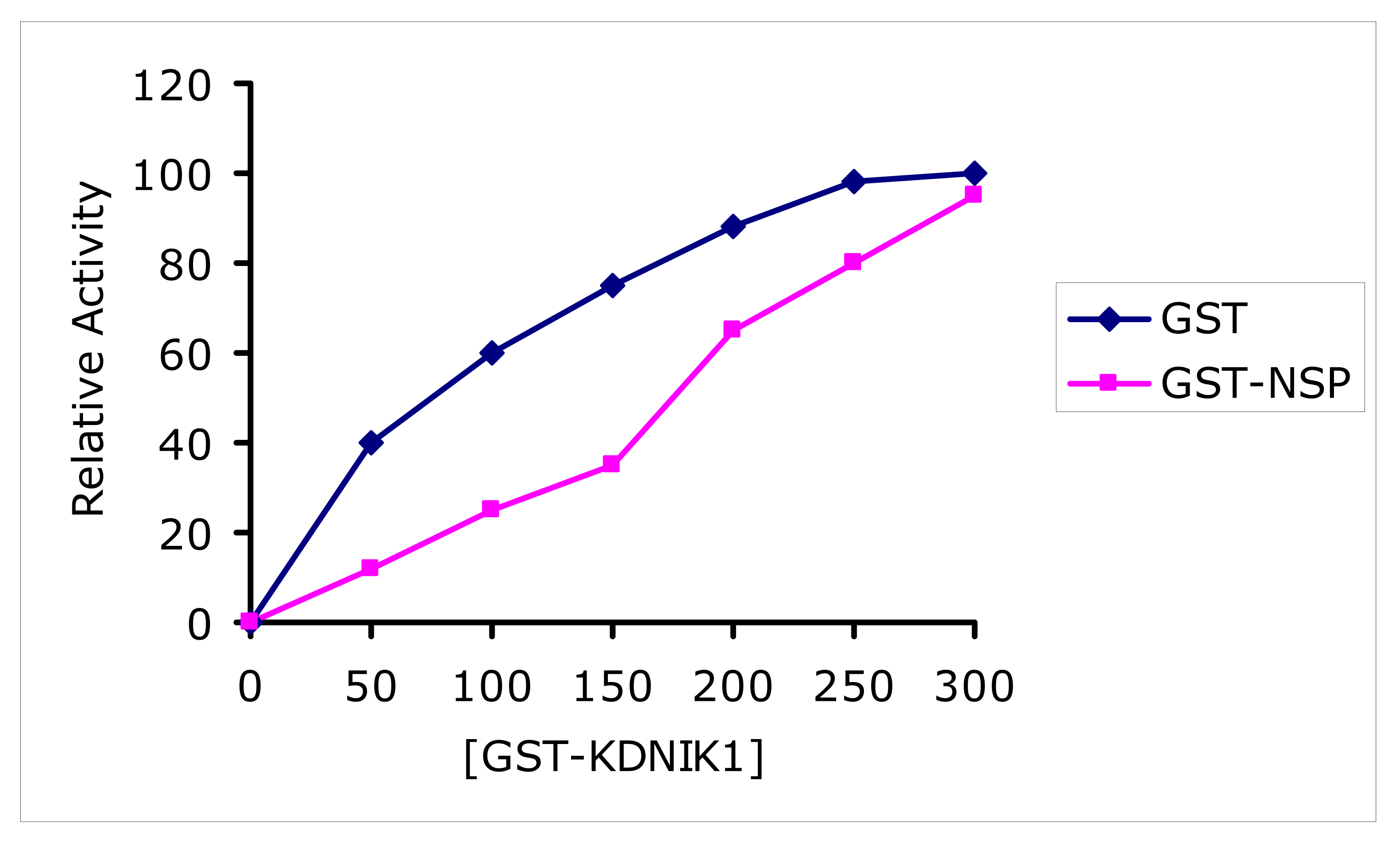

Supplement: Figure S6 — Titration of NSP inhibitor. Increasing amounts of GST-KDNIK1 were incubated with [γ-32P]ATP in the presence of GST (30 ng/µL) or GST-NSP (60 ng/µL). After separation on SDS-PAGE, phosphoproteins were visualized by autoradiography and quantified by phosphoimaging. Relative values of 32P incorporation are the mean of three replicas. (0.45 MB TIF) [file ppat.1000247.s007.tif]

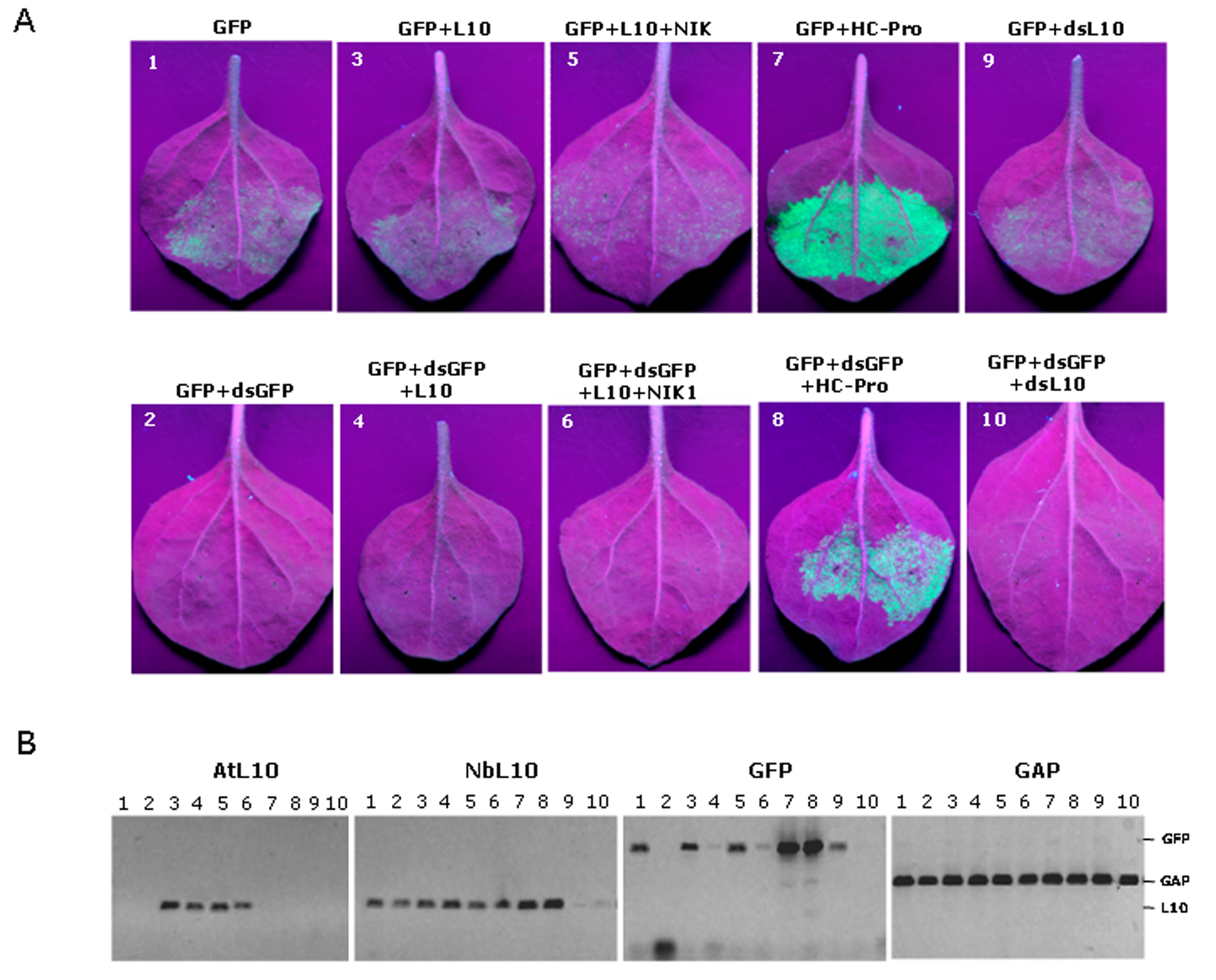

Supplement: Figure S7 — rpL10 is not involved in RNA silencing mechanisms. N. benthamiana leaf tissues were coinfiltrated with Agrobacterium tumefaciens carrying GFP-, dsGFP-, indicated cDNAs-, or inverted repeat RNA-expressing constructs. (A) Photographies were taken under UV lights 5 days postinfiltration. (B) Transcript accumulation in infiltrated leaves. The accumulation of the indicated transcripts was determined by semi-quantitative RT-PCR on RNA extracted from infiltration zones 5 days postinfiltration with gene-specific primers. GAP was used as control. (3.70 MB TIF) [file ppat.1000247.s008.tif]

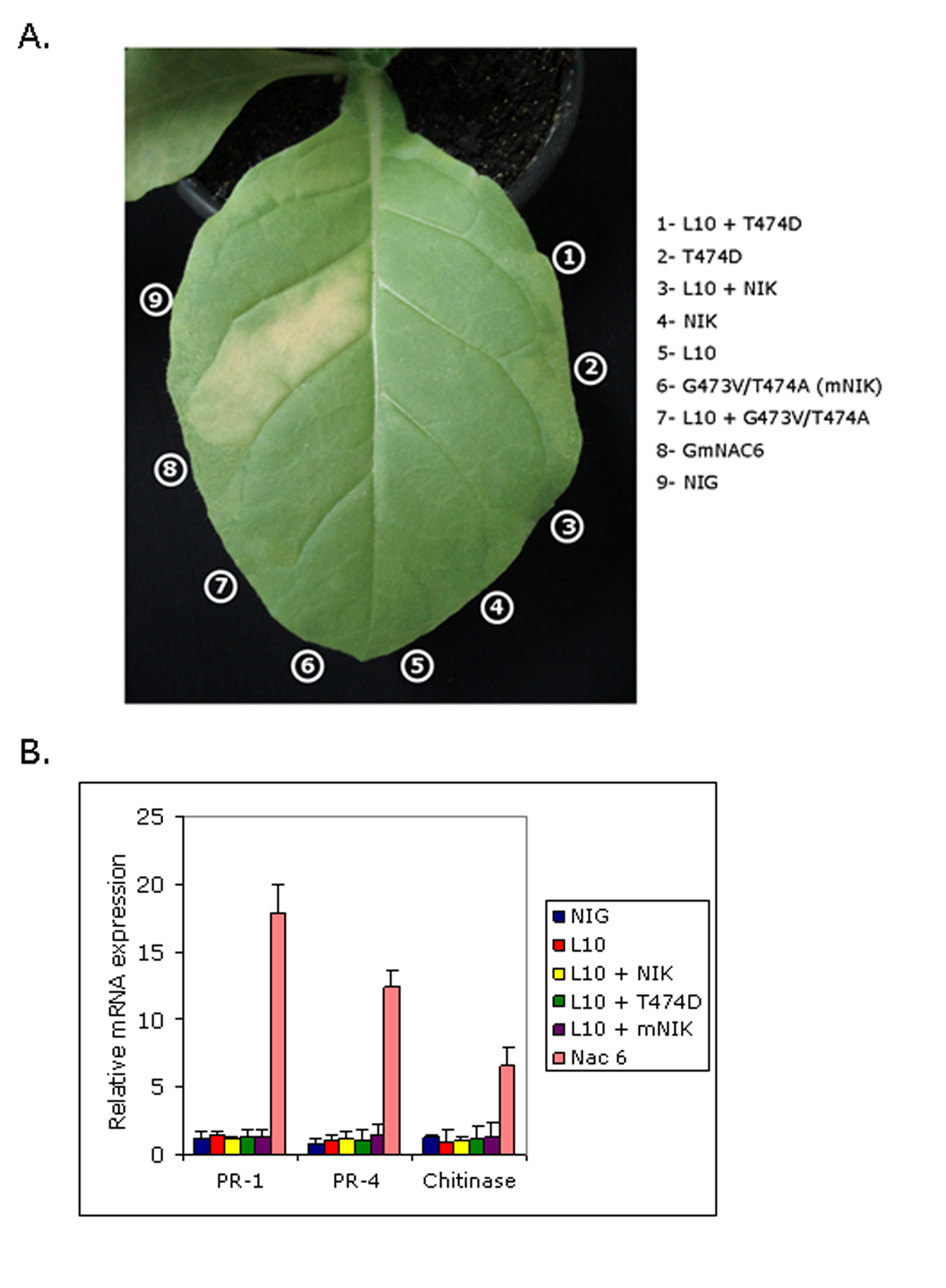

Supplement: Figure S8 — NIK-mediated defense signaling does not induce a hypersensitive response. Tobacco leaf tissues were coinfiltrated with Agrobacterium cultures delivering Ti plasmids expressing rpL10, rpL10 + NIK1, rpL10 + inactive mutant NIK1 (mNIK1), rpL10 +hyperactive mutant NIK1 (T474D), or a control hypersensitive response-inducing soybean NAC6 protein, as positive control, and, as negative control, the NSP-interacting GTPase from Arabidopsis. (A) Leaf necrotic symptoms typical of inducers of hypersensitive response. Leaf sections were agroinfiltrated with the indicated agroinoculum, and pictures were taken 6 days after infiltration. (B) Expression of pathogenesis-related (PR) genes in agroinfiltrated leaves. Two days postinfiltration with the indicated agroinoculum, RNA was extracted from the infiltration zones, and expression of the pathogenesis-related genes PR-1, PR-4, and chitinase was analyzed by RT-PCR. Values are relative to control treatment and represent the mean±SD of three replicates from three independent experiments. (4.72 MB TIF) [file ppat.1000247.s009.tif]
